# Easy and Versatile Synthesis of Bulk Quantities of Highly Enriched ^13^C-Graphene Materials for Biological and Safety Applications

**DOI:** 10.1021/acsnano.2c09799

**Published:** 2022-12-20

**Authors:** Viviana González, Javier Frontiñan-Rubio, M. Victoria Gomez, Tiziano Montini, Mario Durán-Prado, Paolo Fornasiero, Maurizio Prato, Ester Vázquez

**Affiliations:** # Instituto Regional de Investigación Científica Aplicada (IRICA), Universidad de Castilla-La Mancha, 13071Ciudad Real, Spain; § Cell Biology Area, Department of Medical Sciences, Faculty of Medicine, Universidad de Castilla-La Mancha, 13071Ciudad Real, Spain; ⊗ Faculty of Chemical Science and Technology, Universidad de Castilla-La Mancha, 13071Ciudad Real, Spain; † Department of Chemical and Pharmaceutical Sciences, INSTM UdR Trieste, University of Trieste, Via Giorgeri 1, 34127Trieste, Italy; ∇ ICCOM-CNR, University of Trieste, Via L. Giorgieri 1, 34127Trieste, Italy; ∥ Center for Cooperative Research in Biomaterials (CIC biomaGUNE), Basque Research and Technology Alliance (BRTA), Paseo de Miramón 194, 20014Donostia San Sebastián, Spain; ‡ Basque Foundation for Science (IKERBASQUE), Plaza Euskadi 5, 48013Bilbao, Spain

**Keywords:** ^13^C-graphene material, bulk quantities, detection, quantification, safety applications

## Abstract

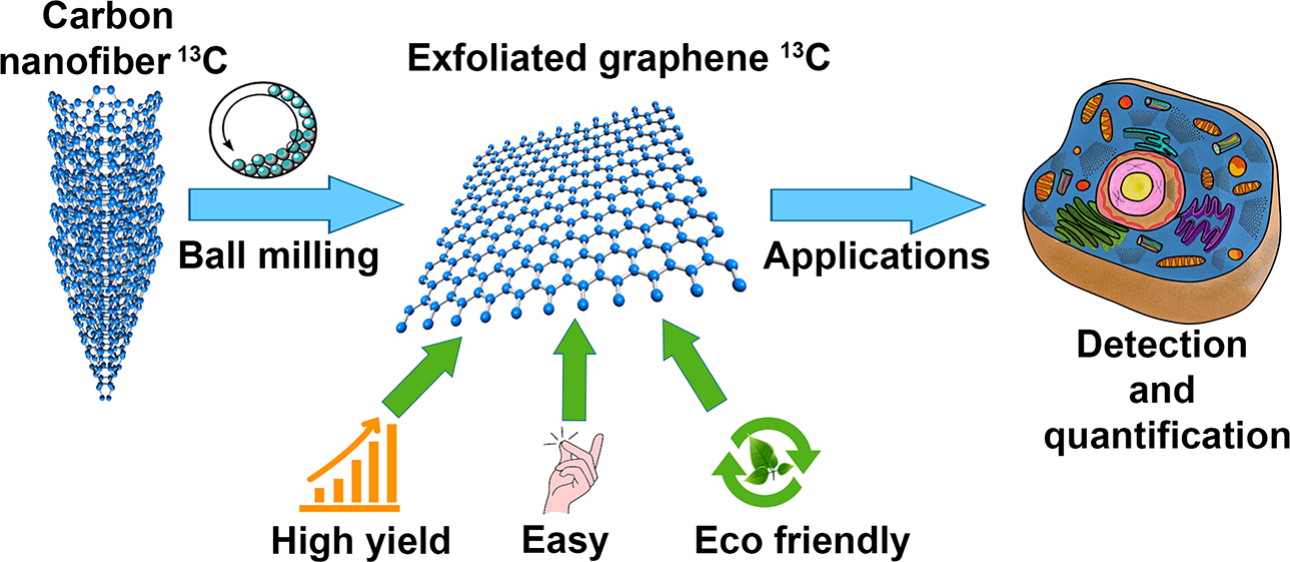

The preparation of bulk quantities of ^13^C-labeled graphene materials is relevant for basic investigations and for practical applications. In addition, ^13^C-labeled graphene materials can be very useful in biological and environmental studies, as they may allow the detection of graphene or its derivatives in cells or organs. In this paper, we describe the synthesis of ^13^C-labeled graphene materials (few-layer graphene, FLG, and graphene oxide, GO) on a tens of mg scale, starting from ^13^C-labeled methane to afford carbon fibers, followed by liquid-phase exfoliation (FLG) or oxidation (GO). The materials have been characterized by several analytical and microscopic techniques, including Raman and nuclear magnetic resonance spectroscopies, thermogravimetric analysis, X-ray photoelectron spectroscopy, and X-ray powder diffraction. As a proof of concept, the distribution of the title compounds in cells has been investigated. In fact, the analysis of the ^13^C/^12^C ratio with isotope ratio mass spectrometry (IRMS) allows the detection and quantification of very small amounts of material in cells or biological compartments with high selectivity, even when the material has been degraded. During the treatment of ^13^C-labeled FLG with HepG2 cells, 4.1% of the applied dose was found in the mitochondrial fraction, while 4.9% ended up in the nuclear fraction. The rest of the dose did not enter into the cell and remained in the plasma membrane or in the culture media.

2D nanomaterials are under intense investigation for applications in many fields of development.^1^ In biological applications, the possibility of these materials to interact, at the nanoscale level, with biological entities gives rise to innovative approaches such as the creation of innovative therapeutic agents or the development of alternative theranostic approaches.^2^ Nevertheless, real-life advances in this area rely on the investigations of the mechanisms with which these materials interact with cells, organs, or other biological compartments and their environment.^3^ Another important issue is that state-of-the-art nanomaterials still generate toxicological concerns:^4^ their end-use and the commercialization of final devices for any particular purpose depend on safety assessments to exclude possible hazards and risks for human health or for the environment, including the determination of exposure limits.^5^ A particular challenge for nanosafety studies is to detect minimal amounts of 2D nanomaterials in complex biological and environmental contexts.^6,7^ For metal-containing materials, the quantification can be achieved relatively easily by standard analytical techniques, such as inductively coupled plasma mass spectrometry (ICP-MS).^8^ However, the quantitative determination of graphene materials is much more difficult, as these materials are mostly composed of carbon atoms and, in lesser amounts, of oxygen and hydrogen atoms, which are the same elements found in the environment or in living systems.^9^

Graphene materials are usually detected by microscopy techniques and Raman spectroscopy. Black, semi-transparent shapes in the transmission electron microscopy (TEM) or confocal microscopy images of biological cross sections are associated with graphene flakes,^10^ while the detection of D- and G-bands in Raman spectra of natural samples is correlated to the presence of the 2D material.^11^ However, even considering only detection without quantification, these techniques are insufficient, mainly because (i) once inside the biological tissues or the natural backgrounds, graphene materials can be degraded^12^ and transformed to structurally and/or chemically different amorphous species which further complicate the recognition, and (ii) using these techniques, it is very difficult to locate the real position of the material within the different biological compartments. For these reasons, some authors have tried to label graphene materials with fluorescent organic labels or radioactive atoms,^4^ but this modification changes the nature/surface of the nanomaterials and can affect their properties and their behavior. Moreover, quenching phenomena and/or detachment of these added moieties from the surface of the 2D nanomaterial can occur, thus making biological and environmental tracing unreliable.^13^

A different approach, very robust and reliable for nanosafety studies and for environmental tracing, is the use of isotope labeling of the graphene material itself.^14^ Some studies have already reported the use of ^14^C radioisotopes to describe the bioaccumulation and biotransformation of graphene in complex biological matrices.^15,16^ However, a simpler concept is the employment of stable isotopes such as ^13^C. ^13^C- and ^12^C-graphene materials synthesized by the same protocol have the same physicochemical properties and intrinsic behavior.^17^ The analysis of the ^13^C/^12^C ratio with isotope ratio mass spectrometry (IRMS) allows the detection and quantification of very small amounts of material in a biological system or compartment, with high selectivity, even when this material has been degraded.^18^ Moreover, although one of the main advantages of the use of radioactive isotopes such as ^14^C is its quantification through imaging techniques, the use of ^13^C magnetic resonance spectroscopic imaging (MRSI) is becoming progressively established.^19−21^ Finally, stable isotope labeling avoids issues associated with the production of radioactive waste and approval of special experimental conditions when using radioactive materials for tracing.^22^

Some authors have already reported the preparation of ^13^C-graphene materials,^23−25^ mostly describing the physicochemical characteristics of graphene prepared by a chemical vapor deposition (CVD) process.^26−34^ The cost of the ^13^C starting materials and the low yield of the synthetic approaches usually prevent the preparation of bulk quantities of ^13^C-graphene materials. However, the enrichment of bulk quantities of GO has also been described with around 7.1 atom% of ^13^C atoms in the final GO, which has made it possible to investigate the bioaccumulation and toxicity of this 2D material in wheat.^35^

In this work, we detail an alternative approach for the production of readily available bulk quantities of ^13^C-graphene materials, both graphene and GO, with different C/O ratios and lateral sizes, suitable for biological studies. The high ^13^C% in the graphene materials (from 95 to 65%) has made it possible to trace graphene materials in individual cell compartments, such as the nucleus and the mitochondria. The protocol described herein will stimulate further investigations to quantify the biodistribution of graphene in different organisms and ecosystems and will permit studies of their biological degradation.

## Results and Discussion

The process leading to ^13^C-graphene starts with the preparation of ^13^C carbon fibers via a CVD process, which are then exfoliated by a standardized ball milling procedure.^36,37^ Ball milling treatments show significantly lower environmental impact for the production of graphene^38^ and can be easily scaled up in order to produce various graphene-related materials.^36,37,39,40^ Our group has previously described that, following these treatments, the preparation of graphene can be achieved by exfoliation of graphite or carbon fibers.^41^ In the present work, ^12^C and ^13^C nanomaterials have been prepared using carbon nanofibers obtained by a CVD process (see Methods). ^12^C nanomaterials have been prepared as controls, and their physicochemical characterization has served to prove that ^13^C labeling does not change the intrinsic structure nor the properties of the materials.

### Synthesis of Carbon Nanofibers

**Figure 1:**
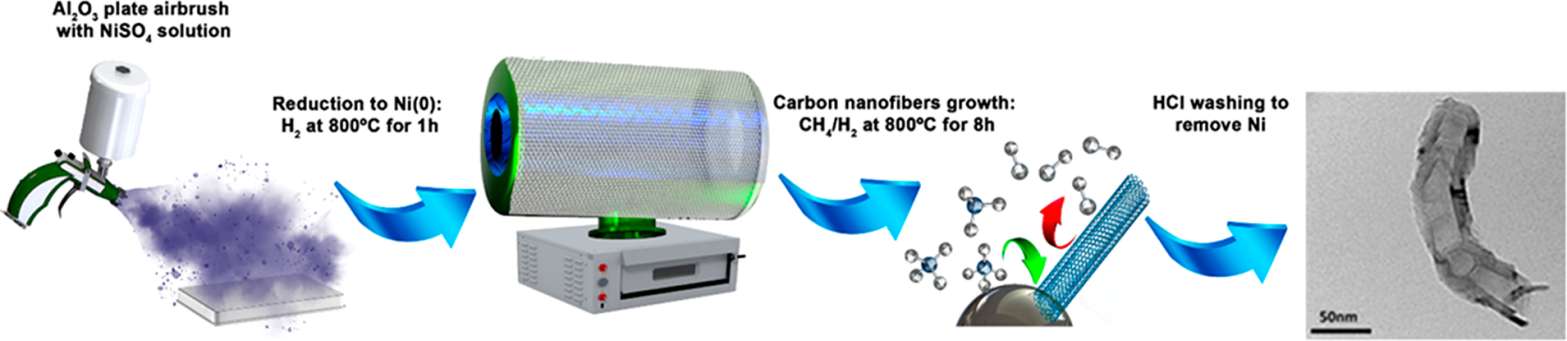
Schematic representation of the synthesis of carbon nanofibers. On the right: typical TEM image of the produced fibers.

Carbon nanofibers were grown by methane decomposition over Ni nanoparticles in a tubular furnace. Figure 1 presents, in a schematic way, the principal steps of the procedure: parameters such as amount of Ni catalyst, concentration of CH_4_ in the reaction mixture, gas flow, and reaction time for nanofiber growth were optimized to maximize the amount of carbon nanofibers obtained from each preparation. In a typical experiment,^42,43^ carbon nanofibers were grown from a H_2_/CH_4_, 30/70 mixture (200 mL min^–1^) over a Ni catalyst (obtained by reduction of NiSO_4_ sprayed over Al_2_O_3_ plates) at 800 °C for 8 h. After cooling under Ar flow, the carbon nanofibers were washed in diluted HCl (to remove accessible Ni), water, and ethanol, finally yielding ^12^C-CNF nanofibers. ^13^C-CNF nanofibers were prepared following the same protocol but replacing CH_4_ with ^13^CH_4_. Fibers prepared in this way showed high quality by TEM. However, to eliminate any trace of nickel metal, the nanofibers were further graphitized at 2700 °C under helium atmosphere for 2 h with 0.5 bar overpressure.^41^ The ^12^C and ^13^C graphitized carbon nanofibers were named as ^12^C-GCNF and ^13^C-GCNF, respectively. All samples were characterized using Raman spectroscopy, thermogravimetric analysis (TGA), elemental analysis, transmission electron microscopy (TEM), and X-ray powder diffraction (XRD).

**Figure 2:**
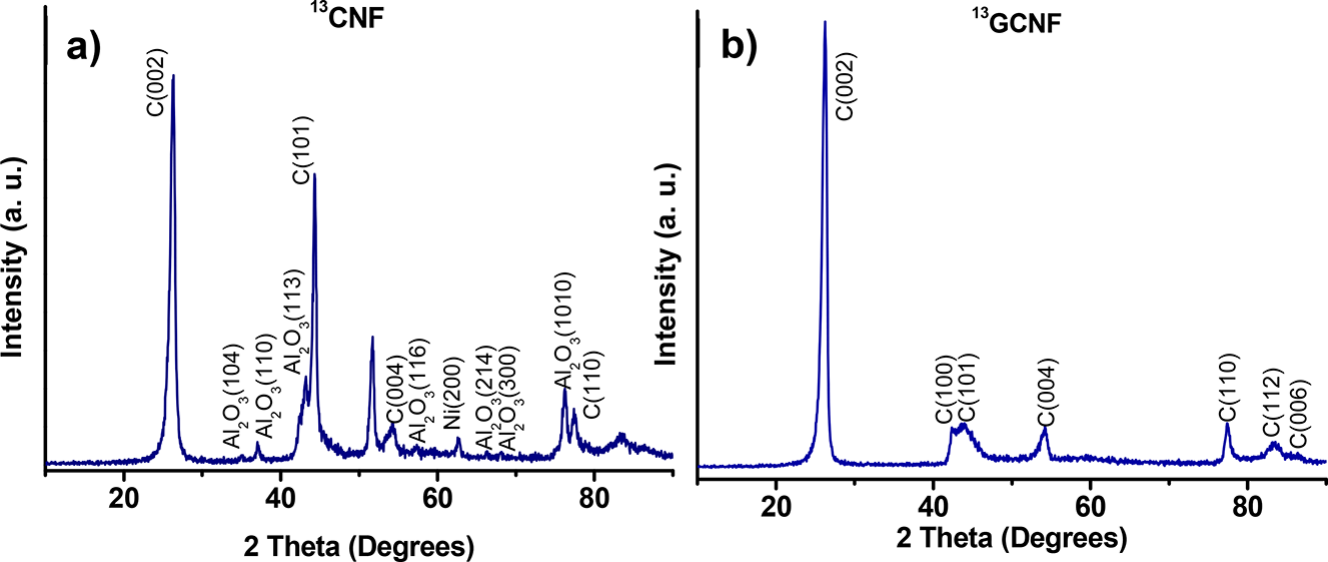
XRD spectra of (a) ^13^C-CNF and (b) ^13^C-GCNF.

Figure 2 shows the XRD spectra for ^13^C-CNF and ^13^C-GCNF in which the two peaks, at 2θ = 26.39° and 42.44°, can be assigned as typical graphitic 002 and 101 planes, respectively. Through the 002 peak intensity plane, it is possible to determine the degree of crystallinity in carbon samples, using eq 1:^44^
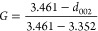
1where 3.461 Å and 3.352 Å correspond to the *d* spacing for a fully turbostatic disordered and highly oriented pyrolytic graphite, respectively.

In general, graphitized nanofibers show a higher degree of crystallinity, determined by the *d*_002_ spacing peak (Table 1). Moreover, carbon nanofibers showed a high quantity of residual metal related to their synthesis. However, after graphitization, no metal residue was observed in the XRD spectra (Figure 2 and S1). The corresponding values for all the parameters obtained through XRD are shown in Table 1 using the peak (002) of carbon in all the different samples.

**Table 1:** Different Parameters Obtained from XRD for ^12^C-CNF, ^12^C-GCNF, ^13^C-CNF, and ^13^C-GCNF

sample	2θ (deg)	*d* (Å)	*G* (%)
^12^C-CNF	26.40940	3.37082	82.73
^12^C-GCNF	26.42480	3.36889	84.50
^13^C-CNF	26.29798	3.38485	69.86
^13^C-GCNF	26.35370	3.37782	76.31

**Figure 3:**
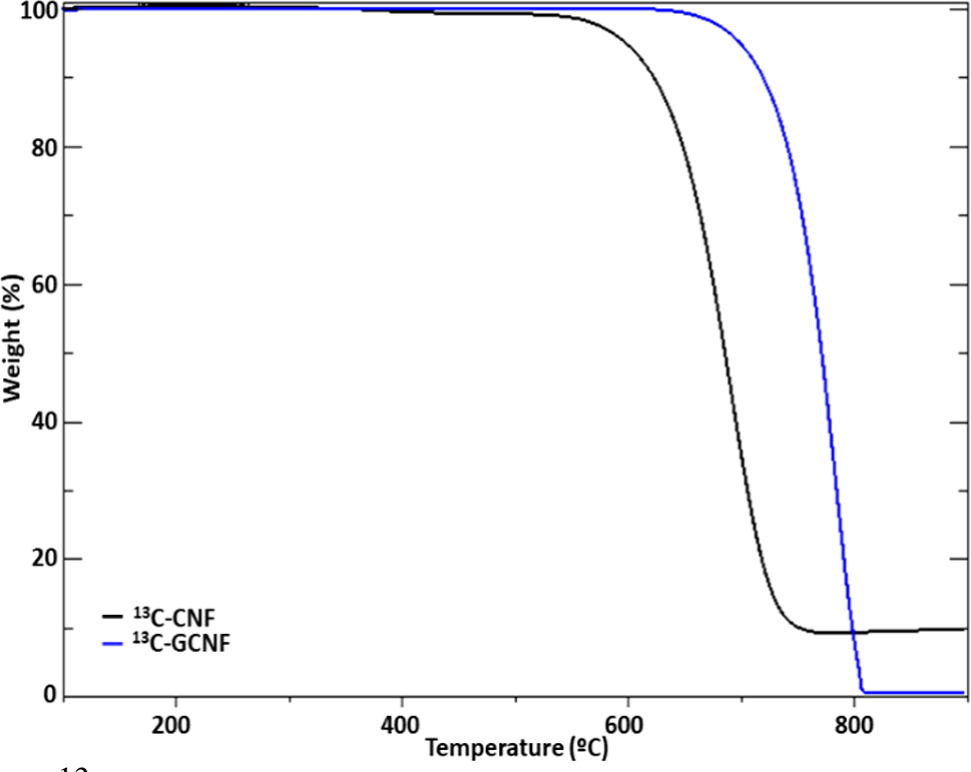
TGA of ^13^C non-graphitized and graphitized carbon nanofibers.

TGAs performed in air atmosphere are shown in Figure 3 and Figure S2 for ^13^C and ^12^C fibers, respectively. As expected, higher thermal stability is observed for graphitized samples (779.93 °C for ^12^C-GCNF, 773.21 °C for ^13^C-GCNF) in comparison to pristine nanofibers (683.95 °C for ^12^C-CNF, 683.52 °C for ^13^C-CNF), corroborating the higher crystallinity in graphitized samples. Moreover, a total weight loss is observed for graphitized samples at 800 °C, while a residual (metallic) mass remains in the non-graphitized fibers (14.12% for ^12^C-CNF, 9.31% for ^13^C-CNF), which indicates the complete removal of all the catalytic nanoparticles in the graphitized nanofibers.

X-ray photoelectron spectroscopy (XPS) was also used to evaluate the type and abundance of oxygen groups in the samples (Figure S3a for ^12^C and Figure S3b for ^13^C). The C 1s and O 1s core-level spectra were fitted with Gaussian–Lorentzian (90G/10L) peaks to give separate main components.

The components of C 1s at different binding energies are 284.5 eV (sp^2^ carbon bonds), 286.4 eV (sp^3^, C–O–C bond), 287.8 eV (C=O bonds), 289.3 eV (C(O)O bonds),^45^ and 291.4 eV (π–π* satellite),^46^ this last one observed in graphitized nanofibers.

On the other hand, the deconvolution of O 1s spectra produces three main peaks in non-graphitized fibers. These peaks at around 531.08 and 532.03 eV are generally assigned to C=O (in either carbonyl or carboxyl groups),^47^ and that at 533.43 eV to C–O (singly bonded oxygen).^48^ The graphitization produces in general a reduction in the oxygen content, mainly visible in the C=O peaks.

The increase in intensity of the π→π* shake-up satellite band of graphitic carbon (Figure S4) observed between non-graphitized and graphitized ^12^C and ^13^C carbon nanofibers is due the reduction of defects in the material after removal of oxygen atoms.^49^

**Figure 4:**
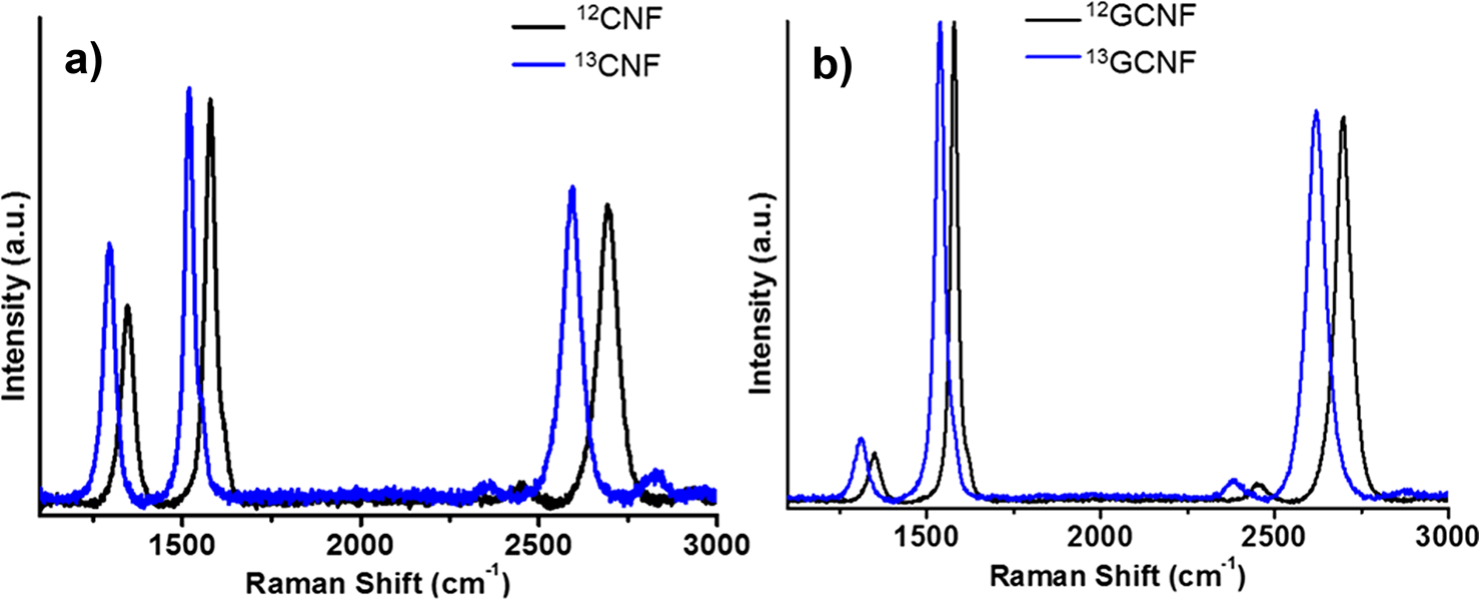
Raman spectra of ^12^C and ^13^C of (a) non-graphitized and (b) graphitized carbon nanofibers.

^12^C-CNF and ^13^C-CNF were also analyzed by Raman spectroscopy (Figure 4), where three bands are observed, corresponding to D, G, and 2D bands. The introduction of heavier ^13^C atoms into the structure leads to downshifts in all the Raman modes. The magnitude of these downshifts depends on the frequency of the mode: those at higher frequencies shift more than modes at lower frequencies, as described by the simple harmonic oscillator model.^33,50,51^

For this reason it is possible to quantify the percentage of enrichment of ^13^C of our sample, following the next equation:^52,53^
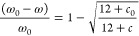
2where ω_0_ is the frequency of the ^12^C sample, ω corresponds to the frequency of the ^13^C sample, *c* corresponds to the concentration of ^13^C in the enriched sample, and *c*_0_ = 0.0107 is the natural abundance of ^13^C.

After applying this equation, we find that before graphitization carbon nanofibers ^13^C-CNF have an enrichment of 95.48 wt% (1519.89 cm^–1^). After graphitization, this value decreases to 65.10 wt% (1538.56 cm^–1^) for ^13^C-GCNF. These data agree with the results of elemental analysis quantifying ^12^C with 2.82 and 33.12 wt% for ^13^C-CNF and ^13^C-GCNF, respectively. The reason for this exchange in the ^13^C/^12^C ratio is not clear yet and is currently under investigation in our groups. Different graphitization conditions might also be employed to avoid the dilution in ^13^C content.^54^

Raman spectroscopy allows us to study the degree of crystallization and graphitization in carbon structures through the relation between the intensities of the D (*I*_D_), G (*I*_G_), and 2D (*I*_2D_) bands.^55,56^ The lower value of the ratio between *I*_D_ and *I*_G_ intensities after the graphitization process (around 0.10–0.14 for both samples, Table 1) indicates the loss of defects and the increase in the crystallization of the carbon nanofibers.

The percentage of ^12^C in the ^12^C samples observed by elemental analysis was 87.20 wt% for the pristine fibers (^12^C-CNF) and 98.79 wt% for the graphitized ones (^12^C-GCNF), which confirms once more the high quality of the graphitized fibers. Meanwhile, the percentages of H, N, and S are similar in both non-graphitized (0.14 wt% of H, 0.03 wt% for N, and 0.03 wt% for S) and graphitized carbon nanofibers (0.01 wt% of H, 0.02 wt% for N, and 0.02 wt% for S). For ^13^C samples, similar results and percentages of the elements in the samples were found in ^13^C-CNF (0.12 wt% of H, 0.04 wt% for N, and 0.03 wt% for S) and ^13^C-GCNF (0.01 wt% of H, 0.03 wt% for N, and 0.02 wt% for S).

**Figure 5:**
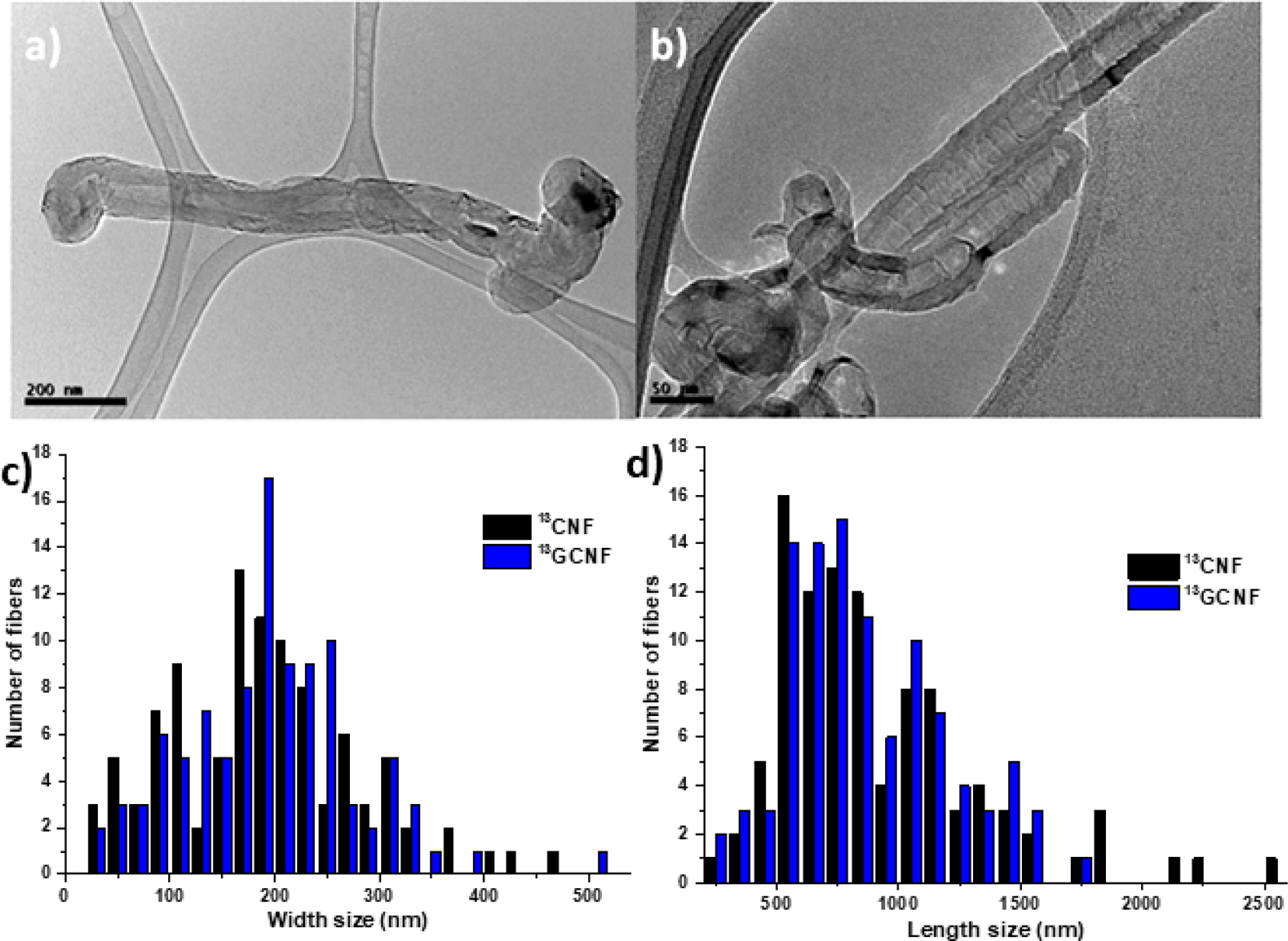
TEM images of (a) ^13^C non-graphitized and (b) graphitized nanofibers. Distributions of (c) diameters (width) and (d) length size of ^13^C carbon nanofibers.

Finally, Figure 5 and Figure S5 show typical TEM images for graphitized and non-graphitized carbon nanofibers, which have homogeneous structure and uniform diameter of around 180 nm with lengths about 800 nm.

All the characterization techniques led us to conclude that both ^12^C and ^13^C fibers show similar structures. Pure graphitized samples exhibit an improvement in their thermal properties, due to a higher degree of crystallinity and to the absence of catalytic nanoparticles.

### Synthesis of Few-Layer Graphene

The exfoliation of carbon nanofibers was performed by using two exfoliating agents, melamine and glucose, through a ball milling treatment for the synthesis of FLG.^36,57,58^ The advantage of using a ball milling treatment is that, by changing the exfoliating agent and the milling parameters, there is the possibility of obtaining graphene flakes with different sizes and of tuning the C/O ratios.^36,39^ Moreover, the process can be considered a green protocol because the exfoliating agent can be recycled and reused in different treatments.

In a typical experiment, the carbon nanofibers and the exfoliating agent are introduced in a stainless-steel grinding bowl with 10 stainless-steel balls (1 cm diameter), and the mechanochemical treatment is performed in a Resch ball mill at room temperature and pressure conditions. After the treatment, the solid is suspended in 20 mL of deionized water, sonicated for 1 min, and then dialyzed to remove the exfoliating agent. The final dispersion is stored for 5 days to allow the remaining carbon fibers to separate from the graphene sample. Finally, the resulting supernatant is separated and lyophilized at −80 °C with a pressure of 0.005 bar to obtain FLG powder.

All the results of the exfoliation of the carbon nanofibers were analyzed mainly by Raman spectroscopy to characterize the quality of the flakes, although the final yield of the process was also considered (Figure S6, Table S2).

In the Raman spectra, there are three principal bands useful to characterize graphene materials: D, G, and 2D bands. The D band is related to the presence of defects, whereas the G band represents the degree of graphitization; therefore, the intensity ratio between these two bands (*I*_D_/*I*_G_) can serve to quantify the density of defects in the graphene material.^59^ Finally, the 2D band can be used to determine the number of layers through its full width at half-maximum (FWHM^60^)—a narrow 2D band indicates a low number of layers.^61^

Considering the information obtained from the Raman spectra and the final yield of graphene obtained, we established a milling time of 2 h at 100 rpm and 5 h at 250 rpm when using melamine and glucose, respectively, as exfoliating agents.

**Figure 6:**
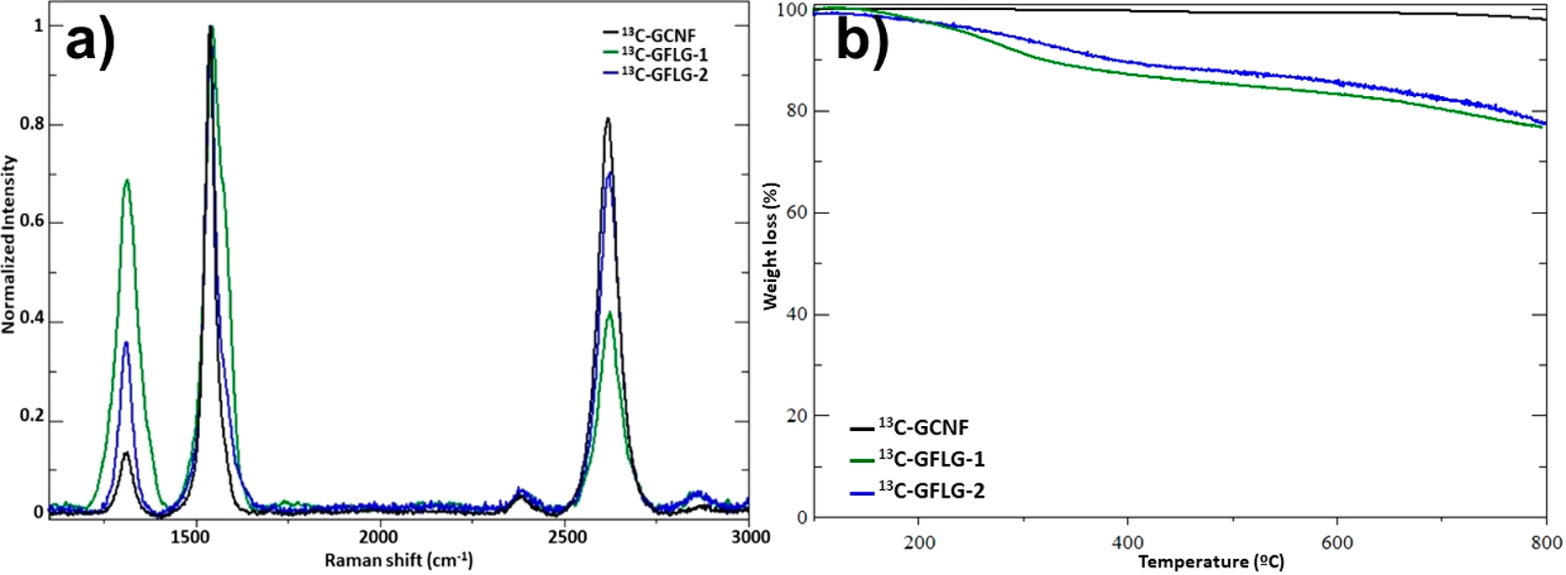
(a) Raman spectra and (b) TGA of carbon nanofibers using graphitized carbon nanofibers and melamine or glucose as exfoliants (^13^C-GFLG-1 and ^13^C-GFLG-2, respectively).

Figure 6 shows the Raman spectra and the TGA of the graphene materials prepared under the optimized conditions, starting with the ^13^C-GCNF (see Figure S7 for ^12^C materials). As already described elsewhere,^36,58^ the ratio *I*_D_/*I*_G_ in the spectra of the graphene materials is lower (Table 2) when using melamine as exfoliating agent in comparison to glucose, which indicates the presence of fewer defects in the former sample. This agrees with the TGA results.

**Table 2:** Raman Spectroscopy Parameters (*I*_2D_/*I*_G_, *I*_D_/*I*_G_ Bands, FWHM, and 2D and G Position Bands) of ^12^C and ^13^C Nanomaterials^a^

sample	*I*_D_/*I*_G_	*I*_2D_/*I*_G_	FWHM (cm^–1^)	2D position (cm^–1^)	G position (cm^–1^)
^13^C-CNF	0.66	0.72	69.08	2593.78	1519.89
^13^C-FLG-1	1.48	0.54	61.60	2586.60	1519.26
^13^C-GCNF	0.14	0.80	68.22	2619.20	1538.56
^13^C-GFLG-1	0.70	0.42	63.77	2622.70	1545.98
^13^C-GFLG-2	0.36	0.81	63.76	2619.02	1539.80
^13^C-GGO	0.86	0.09	–	2634.67	1565.34
^12^C-CNF	0.50	0.76	71.10	2693.80	1578.50
^12^C-GCNF	0.10	0.78	62.91	2696.88	1579.04
^12^C-GFLG-1	1.33	0.57	69.43	2697.74	1583.88
^12^C-GFLG-2	0.55	0.67	62.49	2699.91	1581.54
^12^C-GGO	0.82	0.12	–	2677.62	1584.85

a Samples were carbon nanofibers and graphitized carbon nanofibers (^12^C-CNF, ^12^C-GCNF, ^13^C-CNF, and ^13^C-GCNF), FLG prepared by exfoliation of carbon nanofibers using glucose (^13^C-FLG-1, ^12^C-GFLG-1, and ^13^C-GFLG-1) or melamine (^12^C-FLG-2 and ^13^C-GFLG-2) as exfoliating agent, and graphene oxide prepared from graphitized carbon nanofibers (^12^C-GGO and ^13^C-GGO).

**Figure 7:**
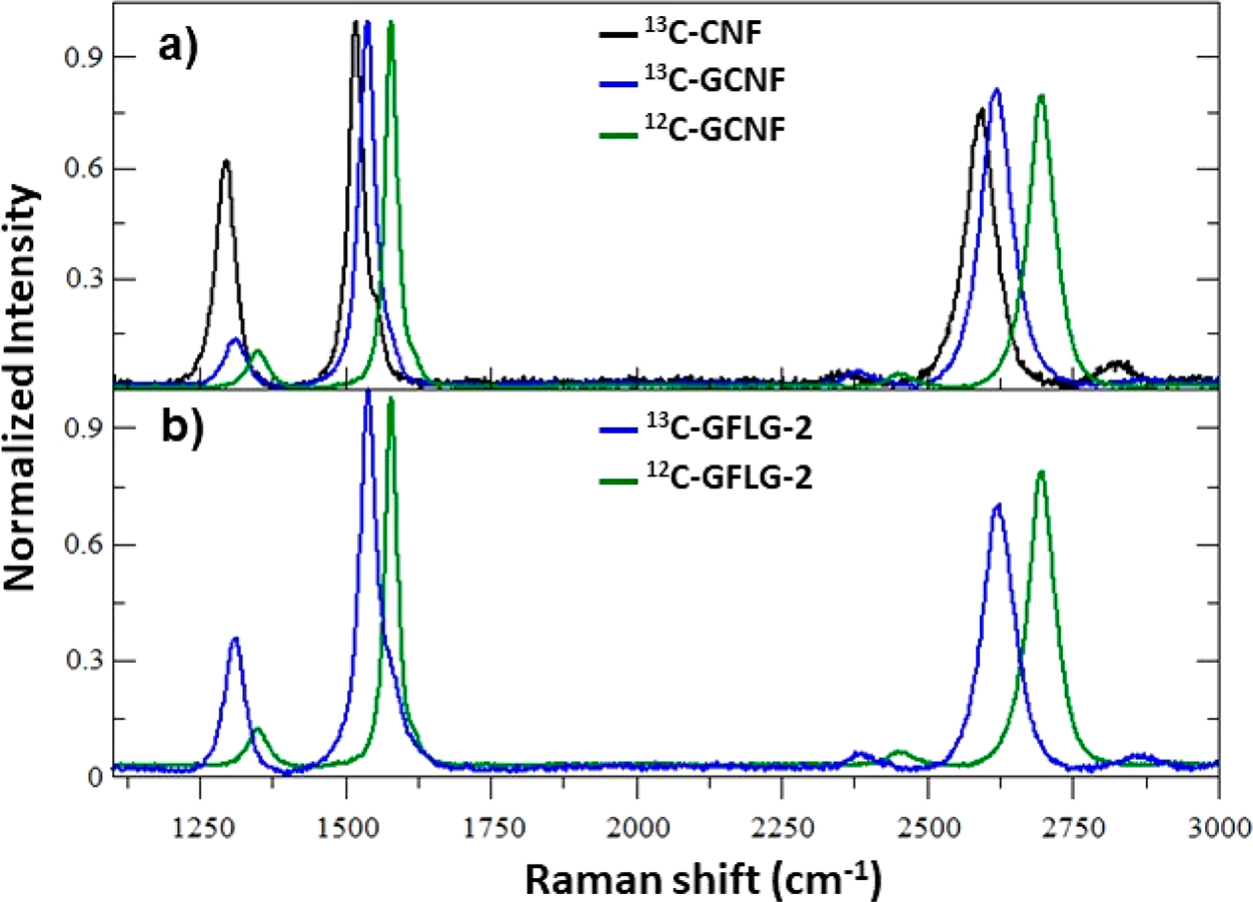
Comparison of Raman spectra of ^13^C and ^12^C nanomaterials: (a) carbon nanofiber and graphitized carbon nanofibers (^13^C-CNF, ^13^C-GCNF, and ^12^C-GCNF) and (b) FLG prepared by exfoliation of carbon nanofibers and graphitized carbon nanofibers using melamine (^13^C-GFLG-2 and ^12^C-GFLG-2).

Figure 7 shows a comparison between the Raman spectra of the different fibers and FLG prepared using melamine with graphitized (^13^C-GFLG-1) and non-graphitized (^13^C-FLG-1) carbon nanofibers, where the shifts in the Raman bands depend on the percentage of ^13^C as previously described (Figure 7a and data in Table 2). Also, in Table 2, we observe the relationship between the different bands *I*_2D_/*I*_G_, *I*_D_/*I*_G_ and the FWHM for the different ^13^C nanomaterials. It is important to mention that the percentage of ^13^C in FLG is 95.48 wt% for ^13^C-FLG and 65.10 wt% for ^13^C-GFLG (the same value as the starting fibers). Only CVD graphene grown on a substrate has been described in the literature with such a high ^13^C percentage.^26,28−30,32−34,50,62^ Other papers describe the synthesis of GO in which the amount of ^13^C is only around 7%.^35^ Our values are much higher in comparison to the data found in the literature for ^13^C-graphene materials prepared on a mg scale, and it is also important to note the high yield in which the material is obtained along with the possibility of recycling the non-exfoliated fibers to produce other batches of ^13^C-FLG.

Raman spectra were used to analyze the different characteristics of the ^13^C-graphene materials (Figure S7a for ^12^C and Table 2). In general terms, all the experiments related to the exfoliation of graphitized carbon nanofibers have shown a small width of FWHM in the 2D band and a *I*_2D_/*I*_G_ ratio lower than 1, which corresponds to FLG. As already commented, lower values of *I*_D_/*I*_G_ are observed when using melamine as exfoliating agent, indicating a less defective graphene in the case of melamine.^36,39^ However, it should be emphasized that the exfoliation with glucose offers the advantage of using a non-toxic and environmentally friendly agent.

TGA was used to check the thermal stability of all samples of graphene (Figure 6 and Figure S7b for ^12^C) under a nitrogen atmosphere. ^12^C-GFLG and ^13^C-GFLG show similar behaviors, with a residual loss of 1.8 and 1.9 wt%, respectively. However, the graphene obtained by exfoliation with melamine has a higher thermal stability in comparison with the graphene obtained using glucose as exfoliant agent, ^13^C-GFLG-2 (11.9 wt%) and ^12^C-GFLG-2 (14.4 wt%) in comparison with glucose samples ^13^C-GFLG-1 (21.9 wt%) and ^12^C-GFLG-1 (17.2 wt%). This confirms the less defective nature of FLG prepared using melamine as exfoliating agent. All these values are due to pyrolysis of the residual oxygen groups on the graphene surface.^63^

**Figure 8:**
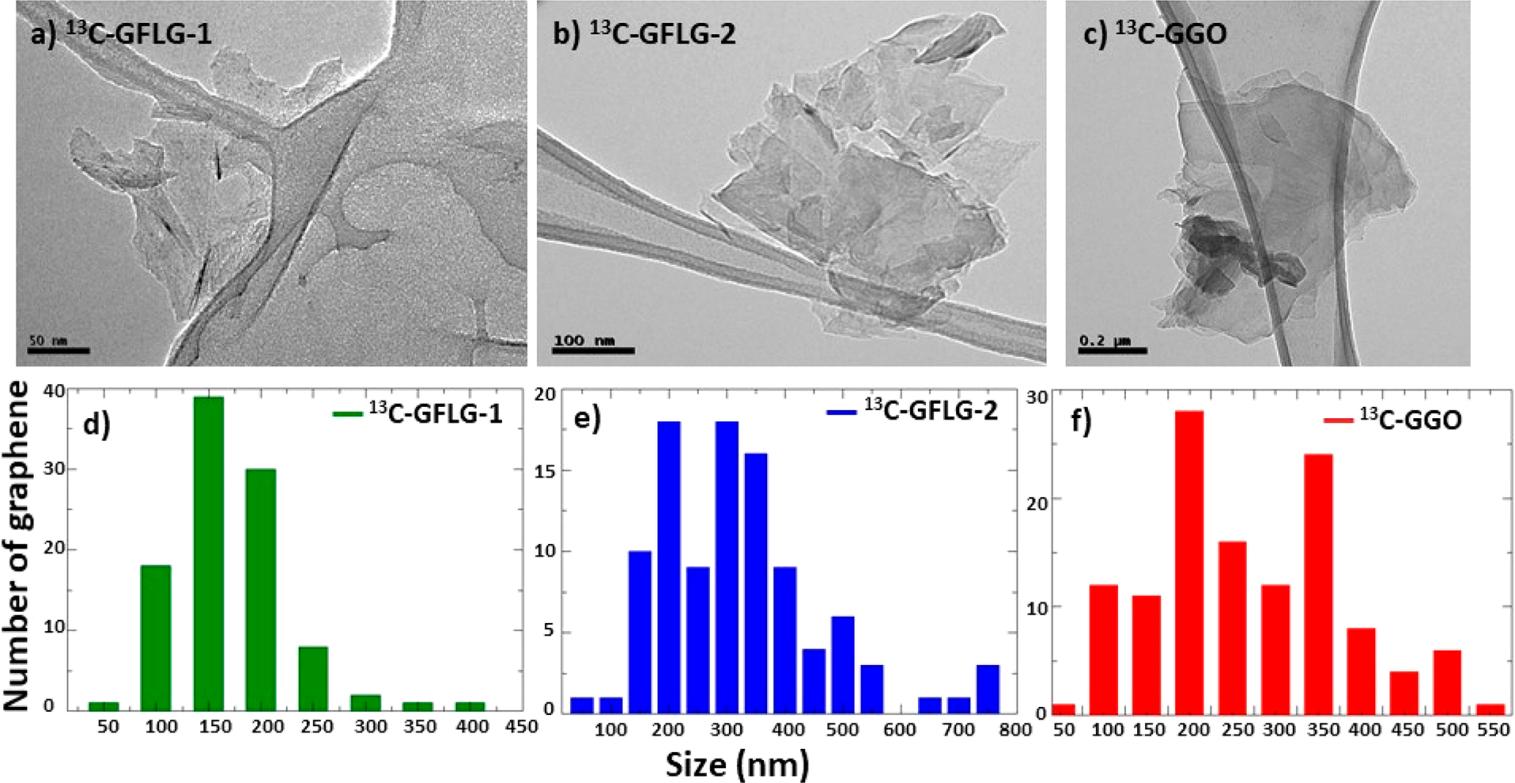
TEM images and size distribution of graphene obtained by exfoliation of the ^13^C graphitized carbon nanofibers: (a, d) ^13^C-GFLG-1, (b, e) ^13^C-GFLG-2, and (c, f) ^13^C-GGO.

TEM images and size distributions of graphene obtained by the two methods of exfoliation are shown in Figure 8 for ^13^C and Figure S8 for ^12^C. The size distributions of ^12^C and ^13^C materials are very similar and mainly depend on the exfoliating conditions, larger flakes being observed when using melamine as exfoliating agent.

**Figure 9:**
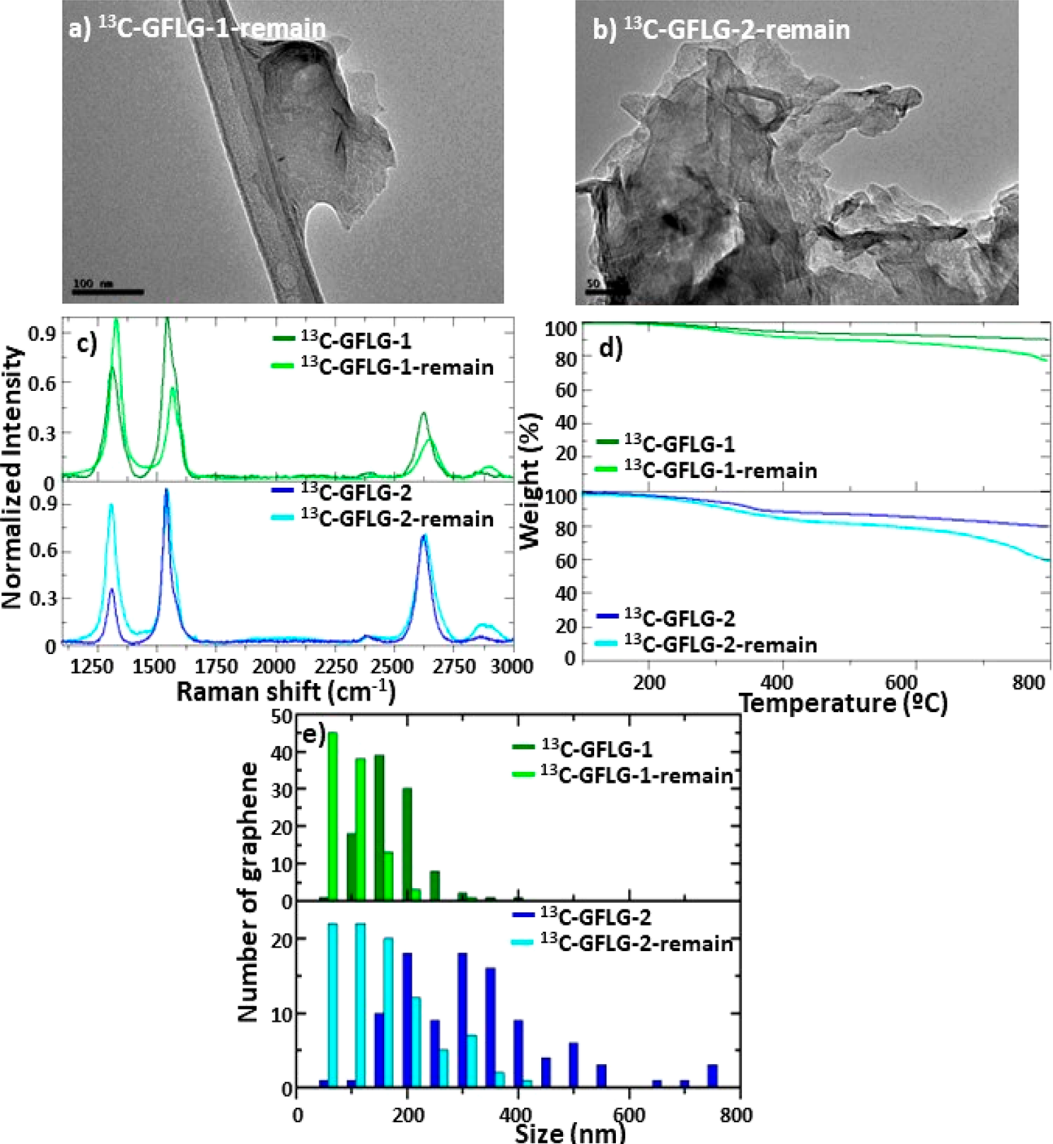
Comparison of characterization results obtained for FLG materials prepared from pristine graphitized carbon fibers (^13^C-GFLG-1 and ^13^C-GFLG-2) and recovered graphitized carbon fibers (^13^C-GFLG-1-remain and ^13^C-GFLG-2-remain): (a, b) TEM images, (c) Raman spectra, (d) TGA, and (e) size distribution of FLG flakes.

Although the yield of the preparation of FLG, considering the recovery of the non-exfoliated fibers, is quite high (around 44%), we have also exploited the possibility of resubmitting these fibers to a new exfoliation process. Figure 9 shows the characterization of the FLG materials when using glucose and melamine as exfoliating agents in a second exfoliation procedure, ^13^C-GFLG-1-remain and ^13^C-GFLG-2-remain, respectively. In general, we found smaller flakes, but the TGA shows a similar thermal stability compared to the previous results of FLG, which corroborates the absence of oxidation during the treatment. Moreover, in Raman spectra, it is possible to observe an increase in the *I*_D_/*I*_G_ ratio that is related to the decrease in the flake sizes.

### Synthesis of Graphene Oxide

GO was prepared using a modified Hummers method starting from ^12^C-GCNF or ^13^C-GCNF as starting material^64^ (see Methods).

**Figure 10:**
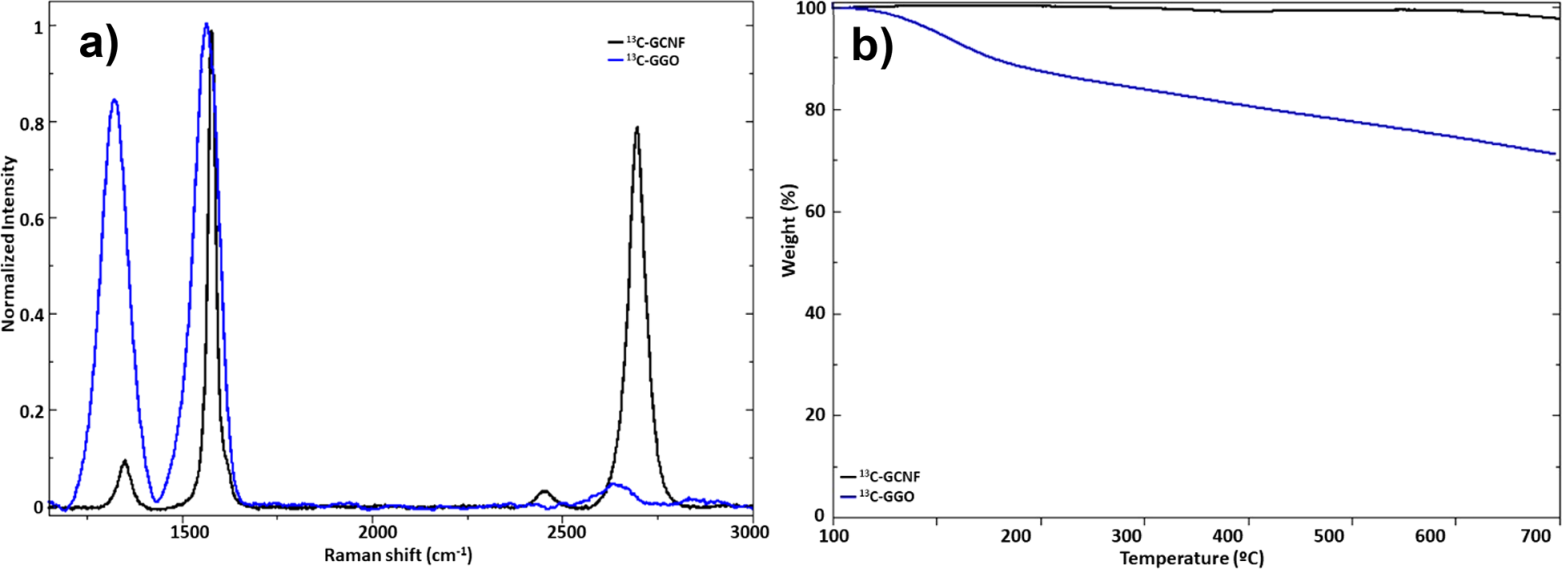
(a) Raman spectroscopy and (b) TGA of ^13^C-graphene oxide (^13^C-GGO).

Raman spectroscopy was used to follow the process from graphitized carbon nanofibers to graphene oxide, where it is possible to observe the disappearance of the 2D band in the graphene oxide prepared from graphitized carbon (GGO) for both samples (Figure 10a for ^13^C, Figure S9a for ^12^C, and Table 1), due to loss of interlayer bonding of graphene layers.^65^ It is also possible to observe, for both samples ^12^C and ^13^C, an increase in the D band correlated with the appearance of defects in the structure.

Figure 10b for ^13^C and Figure S9b for ^12^C were used to observe the thermal stability of the GO nanomaterial in a nitrogen atmosphere. In general, TGA analysis shows a low percentage of oxygen groups on the surface compared to other typical GO syntheses, revealing a 31.2% weight loss for ^13^C-GGO.

TEM image and size distribution of graphene oxide are shown in Figure 8 and Figure S8. The medium lateral size is around 220 nm for ^12^C-GGO (Figure S8) and about 240 nm for ^13^C-GGO (Figure 8), again corroborating similar materials from ^12^C and ^13^C fibers.

Finally, the colloidal stability of the ^13^C and ^12^C materials in deionized water was evaluated by UV–vis absorption spectroscopy (Figure S10) at 660 nm for FLG and 386 nm for GO.^40^ Nanomaterial powders were re-dispersed at three different concentrations (0.2, 0.1, and 0.05 mg/mL). The stability of FLG (^12^C-GFLG-1, ^13^C-GFLG-1) increases significantly with respect to that of graphitized carbon nanofibers (^12^C-GCNF, ^13^C-GCNF). Moreover, the stability of graphene oxide (^13^C-GGO, ^12^C-GGO) was compared to that of a commercial source (GO-Antolin). In all the experiments, it is possible to observe a high stability of our ^13^C nanomaterials, which show a minor sedimentation after 48 h (around 25% and 22.5% for ^13^C-GGO and ^13^C-GCNF-1, respectively). Another great advantage of this methodology is the use of glucose as exfoliating agent, which makes the material prepared with the present protocol ideal for biological experiments and applications.

### Solid-State ^13^C Magic-Angle-Spinning Nuclear Magnetic Resonance Spectroscopy (^13^C MAS NMR)

**Figure 11:**
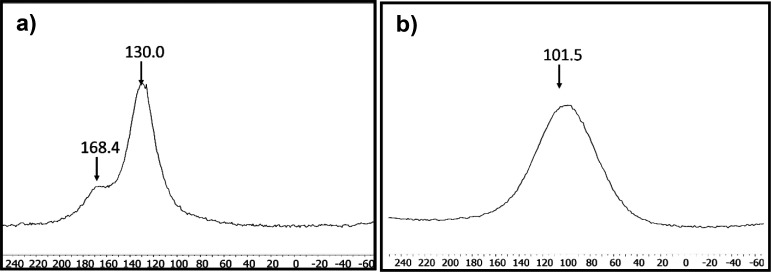
MAS ^13^C NMR spectra for (a) ^13^C-GGO and (b) ^13^C-GFLG-1.

Finally, high-resolution solid-state NMR spectroscopy combined with magic angle spinning (MAS) has been used to characterize ^13^C-GGO and ^13^C GFLG-1, based on the work from Bianco et al.,^66,67^ where MAS NMR played a key role for a comprehensive characterization of functionalized graphene derivatives. The enrichment of graphene with ^13^C enables the structural characterization in shorter times in comparison to the non-labeled sample, where the NMR-active carbon isotope (^13^C) is present naturally at only 1.1%. Figure 11, left, shows the MAS NMR spectrum of ^13^C-GGO, where two peaks are observed. The signal at 168.4 ppm is attributed to the carbonyl ^13^C=O species existing in GO, in agreement with theory and previously reported data, where a peak at 169 ppm was observed.^23,68,69^ The peak centered at 130.0 ppm can be attributed to the aromatic carbon atoms of GO and possibly aromatic C-OH (phenol and/or aromatic diol species) according to previous peak assignment, reported at 129 ppm.^23,68−70^ The absence of peaks at around 60–70 ppm indicates the minor presence of C-OH and epoxy groups attached to aliphatic carbons.^70^ The absence of these aliphatic peaks in the MAS NMR spectrum is in good agreement with the TGA spectrum, which shows the low oxidation degree of this sample (Figure 10b). On the other hand, CP-MAS NMR spectroscopy was also carried out on the ^13^C GFLG-1 sample. The sample could be tuned properly because it is a graphitized sample (absence of metals) in contrast to other works, where electric conductivity caused tuning problems.^67^ The spectrum for ^13^C GFLG-1 shows a single and broad peak at 102.0 ppm that corresponds to sp^2^-^13^C from aromatic entities and conjugated double bonds (Figure 11, right). Due to the lack of functionalization, no other peaks are expected.

The shift to lower chemical shifts (101.5 ppm) compared to GO (130.0 ppm) can be explained due to the absence of oxygen in the ^13^C-sp^2^ surroundings or to magnetic susceptibility effects, knight shift effects, and/or other effects, as previously reported by Bianco et al.^67^ It is consistent with the lower chemical shift values for ^13^C-sp^2^ carbon signals found for graphite nanofibers (^13^C-sp^2^ chemical shift of 80 ppm^71^) and graphite powder (^13^C-sp^2^ chemical shift of 97 ppm^72^) in comparison to oxidized graphene samples.

The bandwidths of the ^13^C-sp^2^ peaks for ^13^C-GGO (10 400 Hz (26 ppm)) and for ^13^C-GFLG-1 (26 000 Hz (65 ppm)) are common values observed for solid-state NMR due to chemical shift anisotropy and other factors.^67,71^ As observed for graphite nanofibers (bandwidth of 90 ppm)^71^ or graphite,^72^ it can be related to the presence of different chemical species with different magnetic susceptibility (heterogeneity in the types of sp^2^ carbons or many non-equivalent ^13^C sites), or due to structural heterogeneity, which could include sheet stacking that could modulate chemical shifts, or even due to the presence of conduction electrons. Notably, the presence of adjacent ^13^C atoms (all at high abundance) also contributes to peak broadening because of their spin–spin couplings.

### Proof of Concept: Detection of Graphene in Subcellular Organelles

Stable isotope labeling of materials is a relevant method to evaluate their environmental impact or their degradation (biostimulation, bioaugmentation, etc.^73^). However, as far as we know, no study has described the distribution, detection, and quantification of graphene materials inside the different cellular compartments. In the present work, our ^13^C-graphene material is suitable to trace graphene derivatives assimilated by cells, even at very low concentrations. For this purpose, HepG2 cells were exposed to a dispersion of ^13^C- and ^12^C-FLG derivatives (10 μg/mL). In order to avoid any effect produced by metal or melamine traces, graphitized FLG samples prepared using glucose as exfoliating agent were used (GFLG-1), even though these materials present a lower percentage of ^13^C (63 vs 95%).

**Figure 12:**
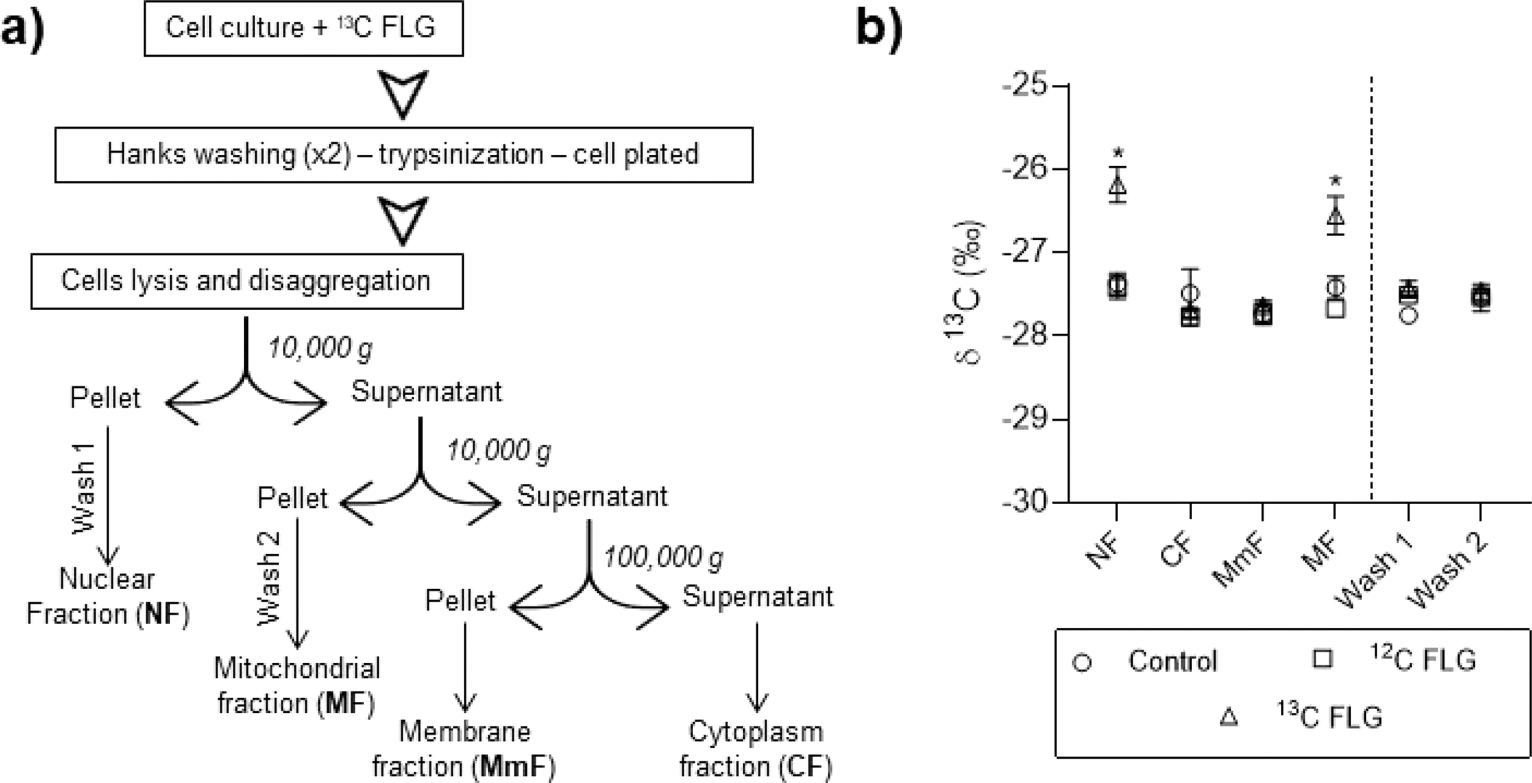
(a) Subcellular fractionation at serial centrifugation steps and (b) their comparison.

After 7 days of incubation, a subcellular fractionation was performed by means of serial centrifugation steps (Figure 12a). We were able to separate fractions including the nucleus (NF), cytoplasm (CF), membranes (MmF), and mitochondria (MF). To ensure that the FLG present in the medium does not contaminate the different fractions, the cells were washed twice, trypsinized, and seeded for 24 h before starting the fractionation process. Moreover, and to completely exclude the possible contamination of the subcellular fractions with graphene materials, for the control experiment, ^13^C-GFLG-1 was added to untreated cells immediately before the fractionation process.

The extracted fractions were lyophilized and analyzed by IRMS. ^13^C NMR spectroscopy was not employed for the analysis mainly because of the presence of an excess (with respect to graphene) of organic material in the sample that also contains ^13^C (as natural abundance) and, therefore, would also give ^13^C NMR signals in the spectrum that could overlap with the ^13^C-graphene peaks. The results are given in the form of stable carbon isotope ratios (δ^13^C). δ^13^C values of the different subcellular fractions can be found in Table 3, and they are plotted in Figure 12b for comparison. δ^13^C values of cytoplasm and membrane fractions were similar in all cases, while nuclear and mitochondrial fractions of cells treated with ^13^C-GFLG-1 were enriched in ^13^C by around 1‰ relative to both control and ^12^C-GFLG-1-treated cells. It is important to note that values found in the buffer obtained from the washing steps of nuclear and mitochondrial fractions (wash 1 and wash 2, respectively) were similar in all the cases, which excludes contamination. Additionally, plasma membrane fractions, extracted in the trypsin part, present an enormous increase in ^13^C (Table 3), corroborating the fact that graphene materials in the culture media and those attached to the plasma membrane were removed from the cellular environment.

**Table 3:** δ^13^C Values (‰, with SD in Parentheses) and %C of the Different Subcellular Fractions

sample		trypsin	NF	wash 1	MF	wash 2	CF	MmF
control	δ^13^C	–22.75	–27.37	–27.75	–27.42	–27.54	–27.49	–27.73
		(0.033)	(0.090)	(0.02)	(0.095)	(0.115)	(0.205)	(0.080)
	%C	17.45	38.43	38.63	38.37	39.48	38.48	38.75

^12^C-GFLG-1	δ^13^C	–22.77	–27.42	–27.50	–27.67	–27.53	–27.77	–27.73
		(0.175)	(0.095)	(0.025)	(0.05)	(0.040)	(0.075)	(0.09)
	%C	17.80	38.14	40.76	38.56	38.40	38.07	38.75

^13^C-GFLG-1	δ^13^C	–13.39	–26.18	–27.42	–26.55	–27.45	–27.69	–27.63
		(0.057)	(0.150)	(0.06)	(0.16)	(0.025)	(0.01)	(0.040)
	%C	16.04	38.87	39.08	38.41	38.09	38.81	38.37

To quantify ^13^C-FLG in nuclei and in mitochondrial fractions, δ^13^C values were transformed into the amount of ^13^C-FLG in the different organelle fractions (*m*_^13^C-FLG_) following eqs 1–3. The relation *m*_dry_/*m*_wet_ was 0.1 for nuclei fractions and 0.09 for mitochondria ones, the weights of the lyophilized pellets being around 3 and 3.5 mg for nuclei and mitochondria fractions, respectively. Moreover, cells were treated with a concentration of 10 μg/mL with a total dose of 50 mg. With these data and using eq 4, it is possible to calculate that 4.1% of the applied dose can be found in the mitochondrial fraction, while 4.9% of the dose ends up in the nuclear fraction. The rest of the dose cannot enter the cell and remains in the plasma membrane or in the culture media. These studies and the possibility of detecting graphene derivatives within cellular compartments may help in studying the mechanisms of interaction of these materials at the cellular level. This approach may be relevant for the quantitative analysis of the internalization of glioblastoma multiforme (GBM). We have previously shown using confocal microscopy that GBM can reach mitochondria in epithelial cells.^74^ Other authors mixed GO with FITC-labeled BSA prior to cells’ incubation and then assayed internalization by confocal microscopy, combined with cell flow cytometry and TEM.^75^ TEM has been widely used to evaluate the internalization of GBM in several biological models, in vitro and in vivo,^76−78^ in some other instances combined with Raman spectroscopy.^79^ However, all these approaches are qualitative and just allow one to determine the presence of the material inside the cell or to compare between different experimental conditions. Labeling GBM with ^13^C offers the possibility to study biodistribution and bioaccumulation in vitro and in vivo in a more accurate way, allowing the correlation between the amount of graphene reaching cellular compartments with alterations in these compartments.

## Conclusion

The synthesis of bulk quantities of ^13^C-labeled graphene materials is important for basic studies. But especially the use of graphene in real-world applications makes it increasingly necessary to use methods for the detection of this material and its derivatives in natural matrices, which will favor its traceability and the development of safe and environmentally friendly systems. The present work contributes significantly to this target by describing the synthesis of graphene materials with varying degrees of oxidation and sizes, all enriched with high amounts of ^13^C, that have not been described to date for bulk materials.

The approach is simple and easily scalable, enabling high yields so that materials can be prepared in high enough quantities to be used in a wide range of applications.

^13^C labeling also enables graphene to be detected even after it has been modified or degraded, without the need for D- or G-band Raman detection or microscopy imaging analysis, so it could be used for degradation studies or even for developing occupational exposure limits.

The results suggest that the use of ^13^C materials is a suitable method to assess the biodistribution of graphene in different models and organisms. To date, ^14^C-graphene has been used for such studies, but although it is a valid approach, it generates a number of technical and practical issues due to radioactivity. With the use of ^13^C-graphene, these problems would be avoided, generating an approach that is simpler and applicable in all types of environments. One of the main advantages of using ^14^C is its quantification by imaging techniques. However, the use of MRSI is progressively gaining ground.^19−21^ Combining this technique with the use of ^13^C-graphene would enable real-time biodistribution studies in animal models.

^13^C materials could also be used for bioaccumulation studies in different ecosystems. The use of ^13^C-graphene allows for much simpler and less environmentally invasive experiments than the use of ^14^C. Previous studies with ^13^C-labeled toxic compounds demonstrate the feasibility and applicability of our proposal.^80^

## Methods

### Materials

Unless otherwise noted, materials were purchased from Fluka, Aldrich, Acros, ABCR, Merck, and other commercial suppliers and used as received. ^13^CH_4_ (99% ^13^C) was purchased from SIAD (Società Italiana Acetilene Derivati S.p.A.)

### Equipment

XRD spectra were recorded on a Philips (Panalytical) model using Cu Kα_1_ (1.54056 Å) at 40 kV and 40 mA. Diffraction patterns were collected in a range of 10–90° 2θ.

Raman spectra were recorded on an InVia Renishaw instrument using powder samples with a 532 nm point-based laser with a power density below 1 mW μm^–2^ to avoid laser heating effects. The resulting spectra (with around 30–40 random locations on each sample) were fitted with Lorentzian-shaped bands in each characteristic band of graphene (D, G, and 2D).

TGA was performed on a TA Instruments Q50 instrument at 10 °C min^–1^ under nitrogen or air flow, depending on the sample, from 100 to 800 °C.

TEM was performed on a JEOL 2100 high-resolution transmission electron microscope (HRTEM) at an accelerating voltage of 100 kV using stable dispersions of graphene dip-cast on Lacey copper grids (3.00 mm, 200 mesh), coated with carbon film, for further drying under vacuum.

UV–vis spectra were recorded on a Cary 5000 UV-vis-NIR spectrophotometer with 1 cm quartz cuvettes. Dual beam mode and baseline correction were used throughout the measurements of FLG (660 nm) and GO (386 nm) for 2–48 h at different time intervals. The determination of the concentration of graphene was determined from the optical absorption coefficient at maximum absorbance, using
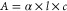
3where *l* (m) is the light path length, *c* (g L^–1^) is the concentration of our material of interest, and α (L g^–1^ m^–1^) is the absorption coefficient, with α = 690 L g^–1^ m^–1^ at 660 nm for FLG and α = 1130 L g^–1^ m^–1^ at 386 nm for GO. The optical absorbance divided by cell length against the concentration exhibited Lambert–Beer behavior.

MAS NMR spectroscopy was performed in a Bruker AV400 wide-bore spectrometer operating in a 9.4 T magnetic field (^13^C, 100.62 MHz) and equipped with a 2.5 mm diameter solid-state probe head. A multinuclear double-channel probe (BL2.5 170-^31^P/^19^F-^1^H) with a spinning speed up to 35 kHz was employed. An amount of around 10 mg of sample was used for each material, ^13^C-GGO and ^13^C-GFLG-1. A pulse sequence of direct excitation on ^13^C via ^1^H decoupling was used. ^13^C MAS NMR spectra of both materials were recorded with a spectral width of 496.9 ppm and an FID size of 2048, with 256 scans, an acquisition time of 0.02 s, a pulse width of 6.2 μs, and a long recycle delay of 180 s based on the previous work reported by Cai et al.^69^ The spin rate for the ^13^C MAS NMR experiment on ^13^C-graphene oxide was 10 kHz and for ^13^C-FLG was 20 kHz. MestReNova v.12.0 was used for data processing.

### ^13^C Quantification by Elemental Analysis Coupled to Isotope Ratio Mass Spectrometry (EA-IRMS)

Lyophilized subcellular fractions were used to detect ^13^C- content by IRMS. Each sample was weighed with a precision balance MX5 (Metler, Toledo) and then encapsuled in tin capsules for isotope analysis. EA-IRMS analyses were performed using a Flash EA1112-ConFloIV analyzer with a MAS 200R carousel autosampler, and a Delta V Advantage (Thermo Scientifics, Bremen, Germany) IRMS was used as the detection system. The elemental analyzer also has a thermal conductivity detector (TCD) for elemental analysis. In the elemental analyzer, the samples pass through a combustion and reduction reactor at 1020 °C, transforming them into CO_2_ and N_2_ gases, which are separated in a chromatographic column at 45 °C. These gases are then transferred to the TCD and IRMS. The carrier gas (helium) was maintained during the analysis at 100 mL min^–1^ and the reference gases (CO_2_ and N_2_) at 250 mL/min. The reference gases were calibrated against internationally certified reference materials supplied by the International Atomic Energy Agency (IAEA). Certified reference materials (IAEA-N1, IAEA-N2, IAEA-CH6, NBS-22, USGS-40, and USGS-41) and laboratory standards were also introduced in each sample sequence.

### Synthesis of Carbon Nanofibers

According to the optimized synthesis conditions, 10 mL of an aqueous solution containing 36.6 mg of NiSO_4_ was slowly sprayed over four alumina plates (75 × 15 mm) heated at 200 °C in order to homogeneously cover their surfaces. NiSO_4_ was sprayed over both sides of the alumina plates. For each batch of carbon nanofibers, 12 spayed alumina plates were placed inside the tubular reactor (inner diameter 45 mm) within a quartz holder properly designed to maximize the exposed area. The tubular reactor was placed within a furnace to homogeneously heat the materials. Prior to each treatment, air was purged from the reactor by an Ar flow (200 mL min^–1^) for 30 min. Subsequently, the furnace was heated at 800 °C (10 °C min^–1^) maintaining the Ar flow in the reactor. After the final temperature was stabilized for 15 min, the gas flow was switched to H_2_ (140 mL min^–1^) to completely reduce the Ni salt, forming Ni nanoparticles on the surface of the Al_2_O_3_ support. After reduction for 1 h, CH_4_ (60 mL min^–1^) was added to the H_2_ flow, reaching a total flow of 200 mL min^–1^ and a linear velocity of ∼12.6 cm min^–1^. The H_2_/CH_4_ mixture was flowed within the reactor for 8 h in order to grow the carbon nanofibers. Then, the furnace was purged by an Ar flow (200 mL min^–1^) for 15 min and cooled. To avoid oxidation and/or partial degradation of the products by accidental exposure to air, the reactor was left to stabilize at room temperature overnight before being opened to remove the Al_2_O_3_ plates with the carbon nanofibers grown on them. The carbon nanofibers were detached from the alumina plates by sonication in EtOH 96% for 1 h and subsequently collected by centrifugation at 4500 rpm for 30 min. Then, the fibers were suspended in aqueous HCl 5% for 24 h, in order to remove the accessible Ni nanoparticles, and washed twice with EtOH 96%. Finally, the material was dried at 60 °C overnight.

The preparation of ^13^C-based carbon nanofibers was performed following the same procedure but replacing CH_4_ with ^13^CH_4_.

### Synthesis of Few-Layer Graphene

Different milling parameters (time and rpm) were used to determine the best conditions of exfoliation of the carbon fibers, using ^12^C-CNF as a model for further implementation with ^13^C nanofibers (see Table S1).

### Synthesis of Graphene Oxide

Graphene oxide was prepared using graphitized carbon nanofibers as starting material. 1 mg of ^13^C-GCNF or ^12^C-GCNF, 1 mL of H_2_SO_4_, and 1 mg KMnO_4_ was stirred for 1 h at room temperature and 6 h at 70 °C. After this time, the material was washed with water until getting a neutral pH.

### Subcellular Fractionation

HepG2 cells were plated into T25 flaks and incubated for 7 d with 10 μg mL^–1^ of ^13^C- or ^12^C-few-layer-graphene materials (^12^C-GFLG-1 and ^13^C-GFLG-1). Then, to ensure the full removal of adsorbed graphene materials, cells were washed with Hanks solution, detached with trypsin, and plated again into T25 flasks for another 24 h. After this time, cells were scraped and lysed using 500 μL of a pH 7.4 fractionation buffer (250 mM sucrose, 20 mM Hepes, 10 mM KCl, 1.5 mM MgCl_2_, 1 mM EDTA, 1 mM EGTA, 1 mM dithiothreitol) supplemented with 1% protease inhibitor cocktail. Lysate was passed through a 25-Ga needle 10 times using a 1 mL syringe and incubated on ice for 20 min. Lysates were centrifuged at 720*g* for 5 min, obtaining the nuclear and cytoplasm/membrane fractions (pellet and supernatant, respectively). The nuclear fraction (NF; pellets) was washed by adding 500 μL of fractionation buffer, resuspended with a pipet, passed through a 25-Ga needle 10×, and then centrifuged again at 720*g* for 10 min. Buffer was removed (wash 1) and NF pellets were resuspended in fractionation buffer. Supernatants were centrifuged at 10000*g*, obtaining the mitochondrial (MF) and cytoplasm/membrane fraction (pellet and supernatant, respectively). MF was washed by adding 500 μL of fractionation buffer, resuspended with a pipet, passed through a 25-Ga needle 10×, and then centrifuged again at 10000*g* for 10 min. Buffer was removed (wash 2) and the mitochondrial pellet was resuspended in fractionation buffer. Finally, the cytoplasm/membrane fraction was centrifuged at 100000*g* for 1 h. Supernatant corresponds to cytoplasm fraction. For membrane fraction, the pellet was washed by adding 400 μL of the fractionation buffer and then re-centrifuged for 45 min. All the fractions were freeze-dried using a Telstar Lyoquest.

As a control, to completely exclude the possible contamination of graphene materials between the subcellular fractions, ^13^C-GFLG-1 was added to untreated cells immediately before the fractionation process (avoiding the incubation step).

### Quantification of ^13^C-Graphene Materials in Subcellular Organelles

To quantify the amount of graphene materials entering inside the different organelles, the extracted pellets were lyophilized to obtain a dry powder. The samples were analyzed by IRMS, and the results were provided as δ values (Table 3). The δ value was converted into a ^13^C/^12^C ratio (*r*) following eq 4, where the (^13^C/^12^C)_standard_ was 0.0111802, the ratio of the Vienna Pee Dee Belemnite (VPDB) standard sample.^25^ ω_^13^C_ (eq 5) is the percentage of ^13^C in mass (total weight of ^13^C atoms/total weight of carbon atoms); thus, ω_^13^C (organelle fraction)_ is the percentage of ^13^C in mass for the organelle fraction from cells exposed to ^13^C-FLG, and ω_^13^C(control)_ is the percentage of ^13^C in mass for the organelle fraction from control cells. ω_^13^C-FLG_ (the percentage of ^13^C in mass in ^13^C-FLG) was calculated from Raman data, obtaining a value of 68.5% for ^13^C-GFLG-1.
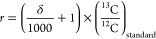
4
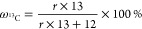
5

The amount of ^13^C-FLG in the different organelle fractions (*m*^13^_C-FLG_) can be calculated from eq 6, where ω_carbon_ is the content of carbon in each fraction obtained by IRMS (Table 2) and *m*_wet_ and *m*_dry_ are the weights of the organelle fractions before and after lyophilizing.
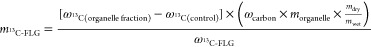
6

The content of ^13^C-FLG in the different organelles was also expressed as percentage of applied dose (%ID, eq 7).
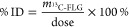
7

## Supporting Information Available

Additional experimental details of the synthesis of graphene, including characterization by XRD, TGA, XPS, and TEM of carbon nanofibers; Raman, TGA, and TEM characterization of ^12^C-graphene; and colloidal stability of the synthesized nanomaterials, including Figures S1–S10 and Tables S1 and S2 (PDF)

## Abbreviations

FLG: Few-layer graphene
GO: Graphene oxide
GGO: Graphene oxide prepared from graphitized carbon
NMR: Nuclear magnetic resonance
TGA: Thermogravimetric analysis
XPS: X-ray photoelectron spectroscopy
XRD: X-ray powder diffraction
IRMS: Isotope ratio mass spectrometry
ICP-MS: Inductively coupled plasma mass spectrometry
TEM: Transmission electron microscopy
MRSI: Magnetic resonance spectroscopic imaging
CVD: Chemical vapor deposition
FWHM: Full width at half-maximum
NF: Nucleus
CF: Cytoplasm
MmF: Membranes
MF: Mitochondria
MAS NMR: Magic Angle Spinning Nuclear Magnetic Resonance
TCD: Thermal conductivity detector
VPDB: Vienna Pee Dee Belemnite
