# Supplementary material for: Easy and Versatile Synthesis of Bulk Quantities of Highly Enriched 13C-Graphene Materials for Biological and Safety Applications
Source: ACS Nano. 2022 Dec 20;17(1):606–20. doi: 10.1021/acsnano.2c09799 (PMC9835986; doi:10.1021/acsnano.2c09799)
Supplement: Supplementary file 1 — nn2c09799_si_001.pdf [file nn2c09799_si_001.pdf]

Supporting Information

Easy and versatile synthesis of bulk quantities of highly enriched 13C-graphene materials for biological and safety applications

Viviana González,[#] *Javier Frontiñan-Rubio,*[#,§] *M. Victoria Gomez,*[#,⁑] *Tiziano Montini,*[†,$] *Mario Durán-Prado,*[§] *Paolo Fornasiero,*[†,$] *Maurizio Prato,*[*,†,‖,‡] *and Ester Vázquez*[*, #,⁑]

# Universidad de Castilla-La Mancha, Instituto Regional de Investigación Científica Aplicada (IRICA), 13071, Ciudad Real, Spain.

§ Universidad de Castilla-La Mancha, Cell Biology Area, Department of Medical Sciences, Faculty of Medicine, 13071, Ciudad Real Spain.

⁑ Universidad de Castilla-La Mancha, Faculty of Chemical Science and Technology, 13071, Ciudad Real, Spain.

† Department of Chemical and Pharmaceutical Sciences, INSTM, UdR Trieste, Via Giorgeri 1, University of Trieste, 34127 Trieste, Italy.

$ ICCOM-CNR, University of Trieste, Via L. Giorgieri 1, 34127 Trieste, Italy.

‖ Center for Cooperative Research in Biomaterials (CIC biomaGUNE), Basque Research and Technology Alliance (BRTA), Paseo de Miramon 194, 20014 Donostia San Sebastián, Spain.

‡ Basque Foundation for Science (IKERBASQUE), Plaza Euskadi 5, 48013 Bilbao, Spain.

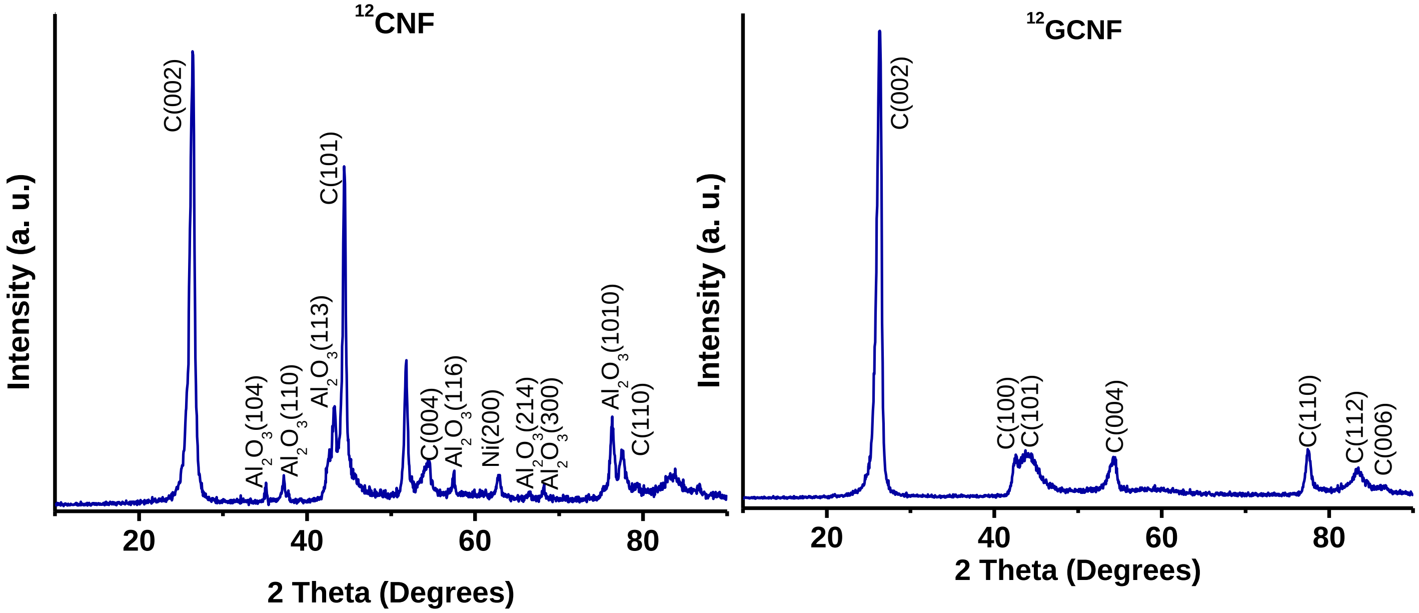

Figure S1. XRD of a) pristine and b) graphitized ^{12}C carbon nanofiber.

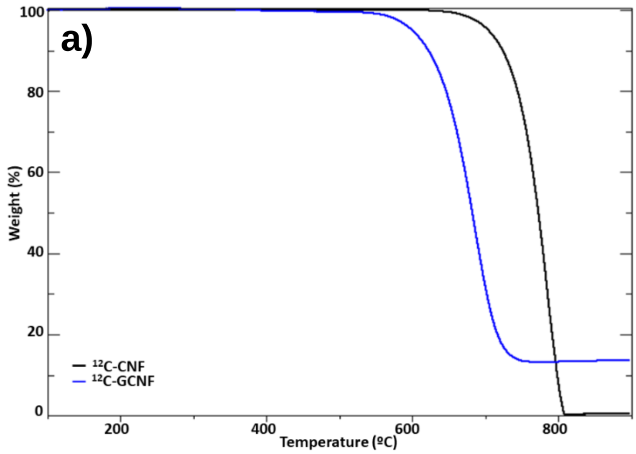

Figure S2.TGA of non-graphitized and graphitized ^{12}C carbon nanofiber.

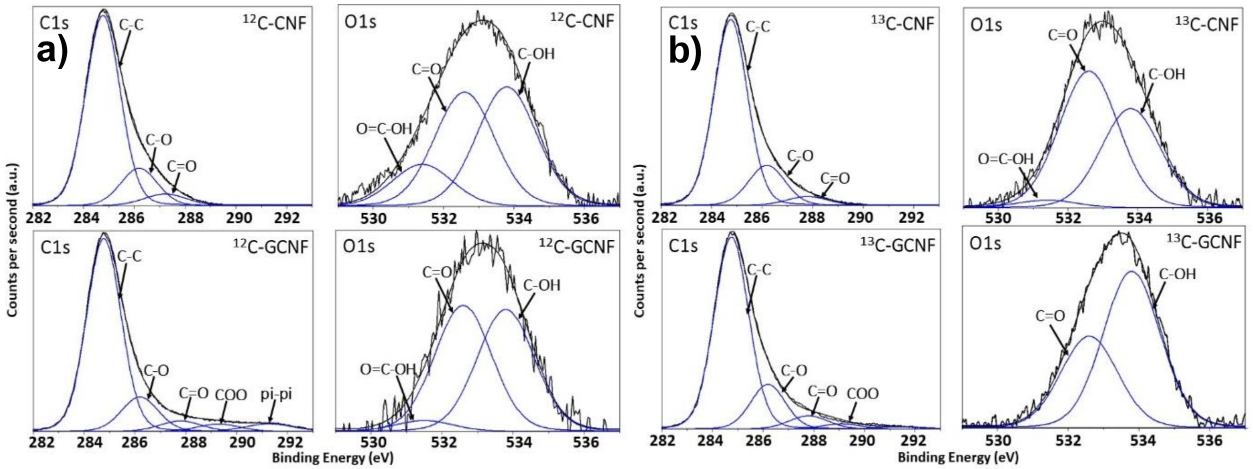

Figure S3. XPS of a) pristine and graphitized ^{12}C and b) ^{13}C carbon nanofiber.

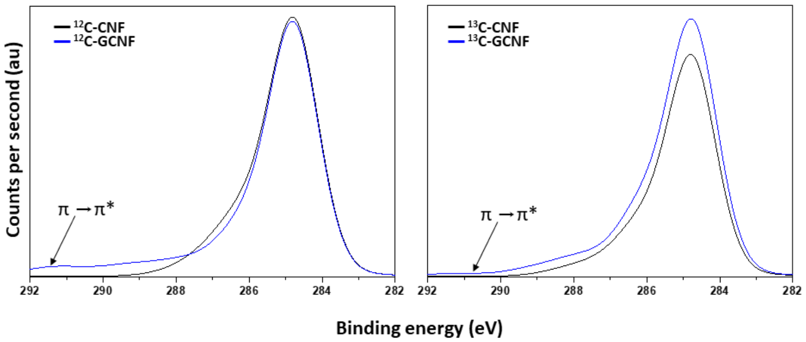

Figure S4.a) XPS of a) pristine and graphitized ^{12}C and b) ^{13}C carbon nanofiber.

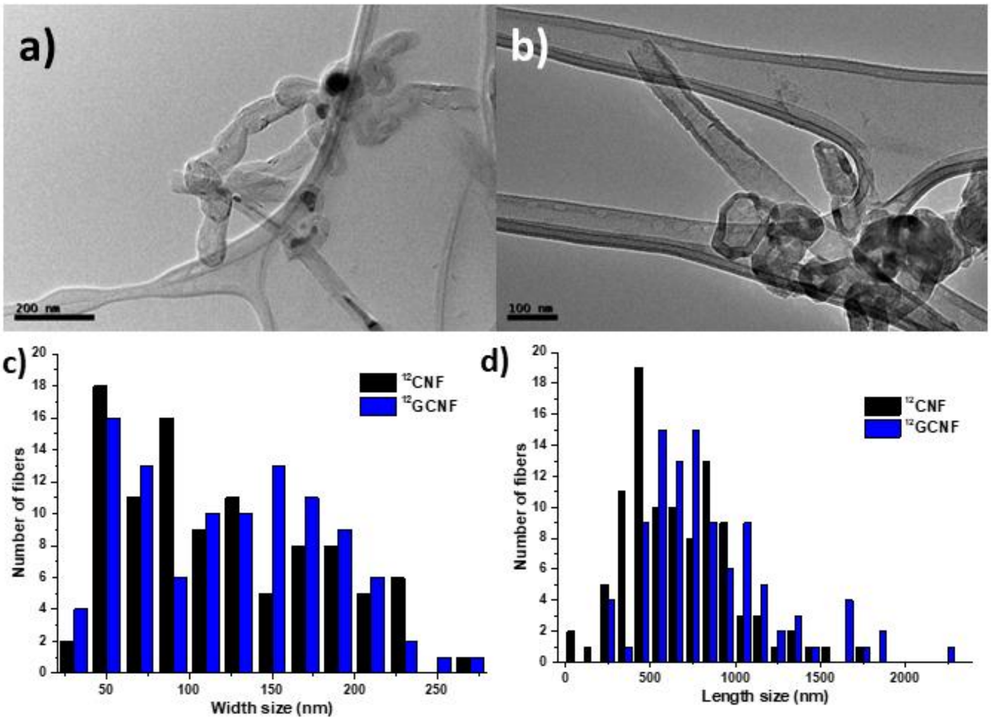

Figure S5. TEM image of a) ^{12}C non-graphitized and b) graphitized nanofiber, c) width and d) length size of ^{12}C carbon nanofibers.

Here, we show the characterization of the exfoliation of non-graphitized ^{12}C-CNF using glucose and melamine at different conditions (30min, 1 and 2hr) for melamine and, (2, 4 and 5hr) for glucose **(Table S1)**.
With this purpose, we use the next system of labelling in this apart:

^{i}C-(G)FLG-n-t

Where:
i is the type of carbon (***^{12}C*** or ***^{13}C***);
G is for ***graphitized*** nanofibers, (without G is used when the starting material is not graphitized)
FLG means **Few Layers Graphene** and,
n depend on the exfoliating agent used (***1 for Glucose*** and ***2 for Melamine***) and,
t represents the time used in the exfoliation process.

Samples	mg Graphite	Exfoliant	mg of Exfoliant agent	Time (hr)	rpm
^{12}C-FLG-2-0.5				0.5	
^{12}C-FLG-2-1	5	Melamine	25	1	100
^{12}C-FLG-2-2				2	
^{12}C-FLG-1-2				2	
^{12}C-FLG-1-4	7.5	Glucose	250	4	250
^{12}C-FLG-1-5				5	

Table S1. Experiments related to exfoliation of carbon nanofibers using glucose and melamine as exfoliating agent.

All the different treatments were analyzed principally by Raman spectroscopy.
In Raman spectroscopy there are three principal bands for graphene nanomaterials (D, G and 2D bands). The D band is related to the defects on the sample, meanwhile the G band accounts for graphitization of the sample, therefore the intensity ratio between these two bands (I_D/I_G) can quantify the density of defects in the graphene flakes.[1, 2] Finally, the 2D band can be used to determine the number of layers of our graphene material through its full width at half maximum (FWHM) [3, 4], a narrow 2D bands indicates a low number of layers[5]
Using glucose as exfoliating agent, the relation of intensities I_{2D}/I_G of the samples corresponding to 2h and 4h of treatment show very similar values, while after 5h there is a slight increment in this value (**Fig. S6**). The I_{2D}/I_G ratio are around 0.5-1 indicating the presence of few-layers graphene.[6]
Meanwhile, the relation I_D/I_G is minimum for the sample of 4h and the maximum value is for 5h of treatment. By other part, with respect to the FWHM, the lowest value corresponds to the synthesis of 4h, however here it is important to mention that this band is not relevant to quantify the number of layers due to the narrow band of the pristine nanofibers. But, in general we can corroborate that 4h of treatment are the best condition for the exfoliation of carbon nanofibers using glucose.
On the other hand, in the synthesis of graphene with melamine (**Fig. S6**), it is possible to

observe similar values of I_{2D}/I_G (around 0.3-0.7) indicating again a structure of few layers graphene in all the samples. The relation I_D/I_G shows lower values that the ones observed in glucose samples, which indicates the presence of few defects in the samples prepared using melamine. Finally, the FWHM, with a range between 52 and 71cm^{-1}, has the lowest value for the sample of 30min indicating a good exfoliation in this treatment .

Moreover, the final yield of whole exfoliation was considered. 4h and 5h of treatment give similar yields. As already discussed 5 h of treatment produce a better exfoliation so we considered this time as the best condition for our experiment. The same idea was assumed in the choice of melamine, where 2hr of synthesis are the best condition to get a major yield in the exfoliation of graphene (**Table S2**).

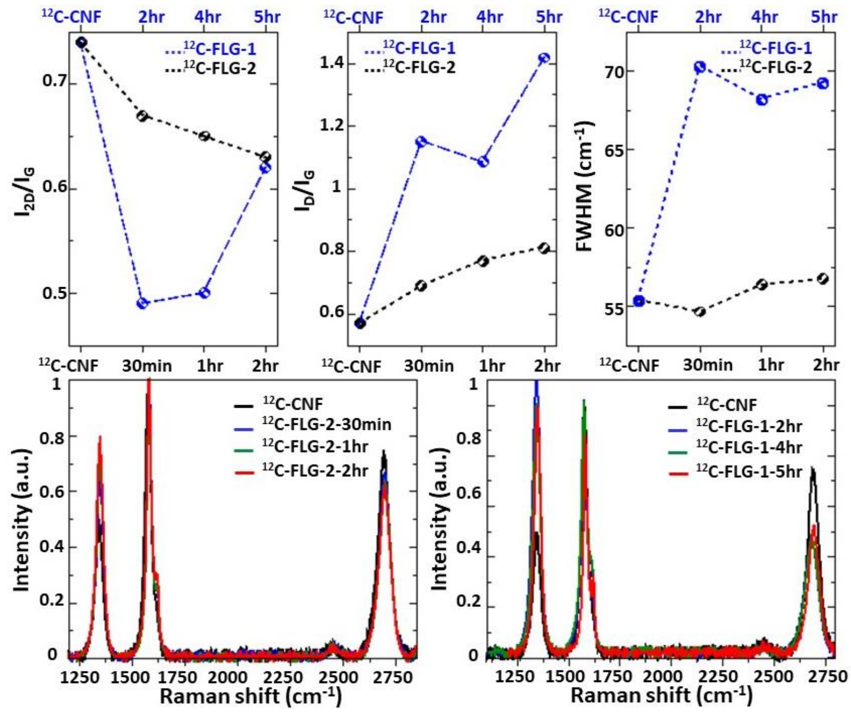

Figure S6. Raman spectra (I_{2D}/I_G, I_D/I_G bands, FWHM, 2D and G position band) of ^{12}C nanomaterials at different times using glucose and melamine as exfoliating agent.

Samples	%Yield of FLG
^{12}C-FLG-2-0.5	39.2
^{12}C-FLG-2-1	41.2
^{12}C-FLG-2-2	43.2
^{12}C-FLG-1-2	40.7
^{12}C-FLG-1-4	41.5
^{12}C-FLG-1-5	45.4

Table S2. Table of the different yields of FLG obtained using the different conditions of exfoliation.

After the observation, we stablished the best condition of exfoliation at 5h for glucose and 2h for melamine, and we tried this conditions to exfoliate ^{12}C-GFLG.

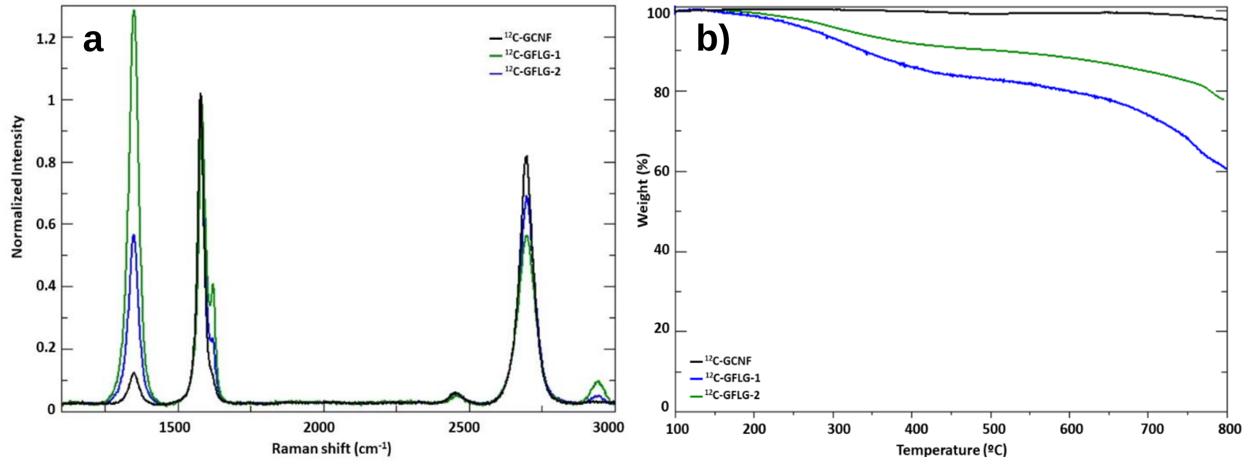

Figure S7. a) Raman spectra and b) Thermogravimetric Analysis of ^{12}C nanomaterials: Graphitized carbon nanofibers (^{12}C-CNF) and, FLG prepared by exfoliation of graphitized carbon nanofibers using glucose and melamine (^{12}C-GFLG-1 and ^{12}C-GFLG-2) as exfoliating agents.

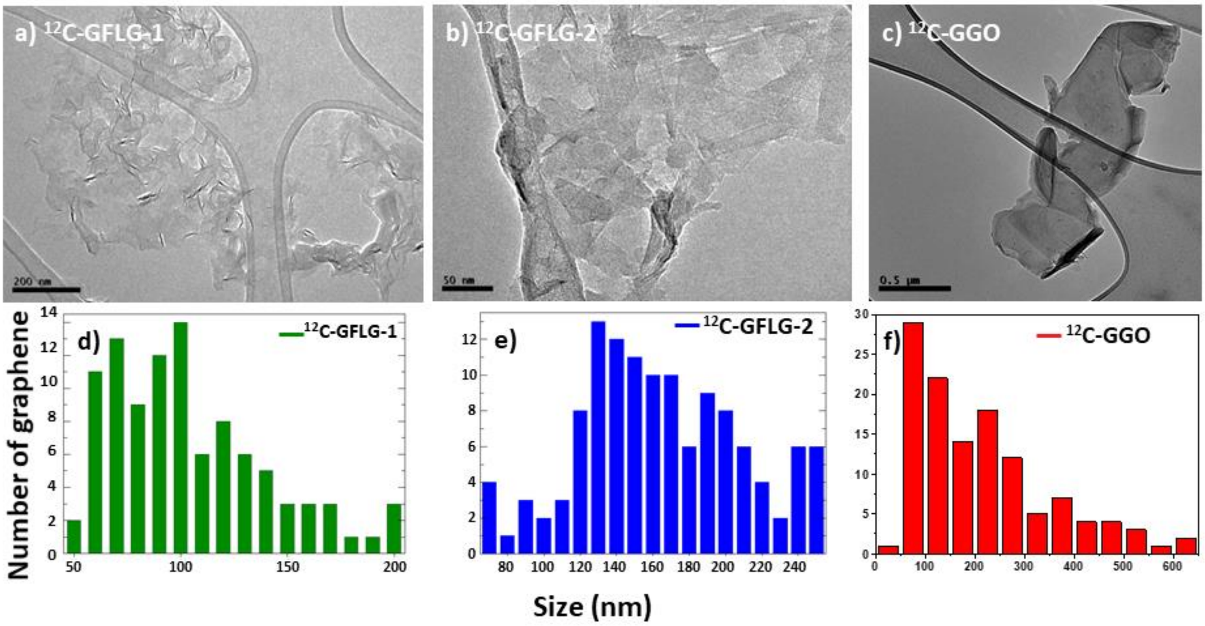

Figure S8. TEM Image and size distribution of graphene obtained by the exfoliation of ^{12}C graphitized carbon nanofibers: a, d) ^{12}C-GFLG-1, b, e) ^{12}C-GFLG-2, c, f) ^{12}C-GGO.

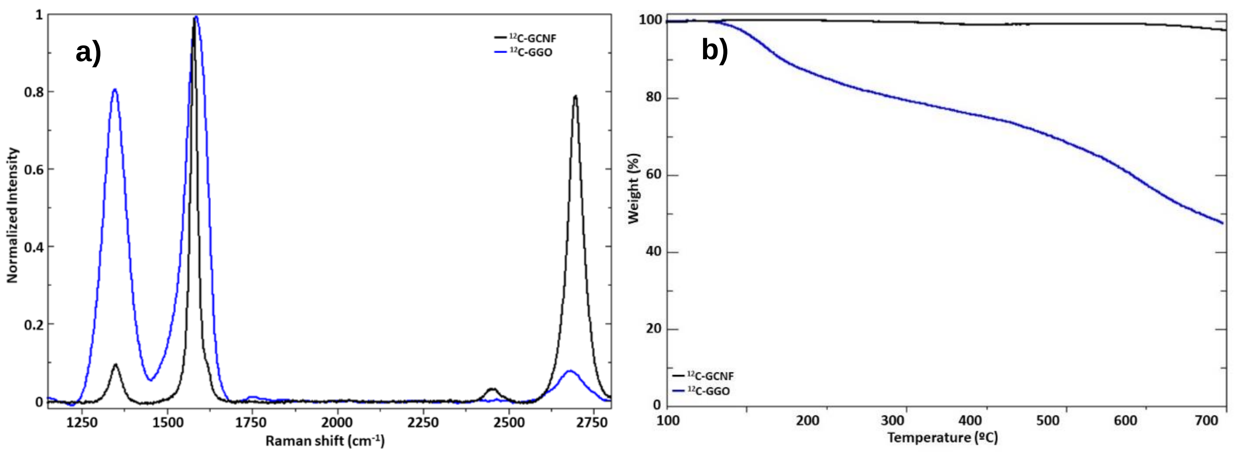

Figure S9. a) Raman spectroscopy and b) Thermogravimetric Analysis of ^{12}C graphene oxide (^{12}C-GGO).

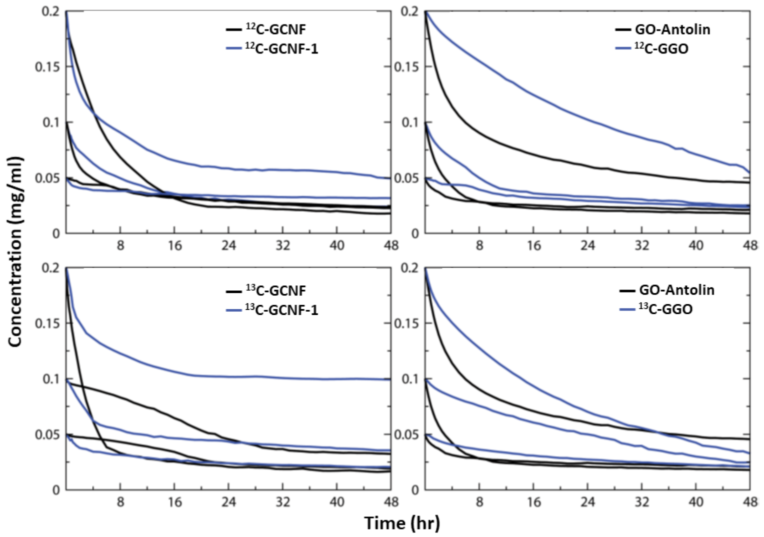

Figure S10. Colloidal stability of the different nanomaterials in water calculated using UV-Vis absorption spectroscopy.

REFERENCES

1. Casiraghi, C.; Hartschuh, A.; Qian, H.; Piscanec, S.; Georgi, C.; Fasoli, A.; Novoselov, K. S.; Basko, D. M.; Ferrari, A. C., Raman Spectroscopy of Graphene Edges. *Nano Letters* **2009,** *9* (4), 1433-1441.
2. Torrisi, F.; Hasan, T.; Wu, W.; Sun, Z.; Lombardo, A.; Kulmala, T. S.; Hsieh, G. W.; Jung, S.; Bonaccorso, F.; Paul, P. J.; Chu, D.; Ferrari, A. C., Inkjet-printed graphene electronics. *ACS Nano* **2012,** *6* (4), 2992-3006.
3. Green, A. A.; Hersam, M. C., Solution Phase Production of Graphene with Controlled Thickness via Density Differentiation. *Nano Letters* **2009,** *9* (12), 4031-4036.
4. Ferrari, A. C.; Meyer, J. C.; Scardaci, V.; Casiraghi, C.; Lazzeri, M.; Mauri, F.; Piscanec, S.; Jiang, D.; Novoselov, K. S.; Roth, S.; Geim, A. K., Raman Spectrum of Graphene and Graphene Layers. *Physical Review Letters* **2006,** *97* (18), 187401.
5. Paton, K. R.; Varrla, E.; Backes, C.; Smith, R. J.; Khan, U.; O'Neill, A.; Boland, C.; Lotya, M.; Istrate, O. M.; King, P.; Higgins, T.; Barwich, S.; May, P.; Puczkarski, P.; Ahmed, I.; Moebius, M.; Pettersson, H.; Long, E.; Coelho, J.; O'Brien, S. E.; McGuire, E. K.; Sanchez, B. M.; Duesberg, G. S.; McEvoy, N.; Pennycook, T. J.; Downing, C.; Crossley, A.; Nicolosi, V.; Coleman, J. N., Scalable production of large quantities of defect-free few-layer graphene by shear exfoliation in liquids. *Nature Materials* **2014,** *13*, 624.
6. Mogera, U.; Dhanya, R.; Pujar, R.; Narayana, C.; Kulkarni, G. U., Highly Decoupled Graphene Multilayers: Turbostraticity at its Best. *J Phys Chem Lett* **2015,** *6* (21), 4437-43.
